# Antitumor Activity of Combined Topoisomerase Inhibition and FLASH Radiotherapy in Head and Neck Carcinoma Biomodels

**DOI:** 10.1002/mco2.70937

**Published:** 2026-09-02

**Authors:** Patrizia Sarogni, Nicoletta Brindani, Valentina Frusca, Melissa Santi, Andrea Cavalieri, Mariagrazia Celentano, Michele Menicagli, Andrea Marranci, Andrea Ghelli Luserna di Rorà, Federico Purificato, Michela Nigro, Noemi Giannini, Alessandra Gonnelli, Giovanni Gadducci, Fabio Di Martino, Fabiola Paiar, Marco De Vivo, Valerio Voliani

**Affiliations:** ^1^ Molecular Modeling & Drug Discovery Lab, Istituto Italiano di Tecnologia Genoa Italy; ^2^ Center for Nanotechnology Innovation @NEST – Istituto Italiano di Tecnologia Pisa Italy; ^3^ Scuola Superiore Sant'Anna Pisa Italy; ^4^ Istituto Nanoscienze‐CNR and NEST Laboratory, Piazza San Silvestro 12 Pisa Italy; ^5^ Center for Instrument Sharing of the University of Pisa (CISUP) University of Pisa Pisa Italy; ^6^ Centro Pisano ricerca e implementazione clinica FLASH Radiotherapy (CPFR@CISUP), Presidio S. Chiara via Roma Italy; ^7^ National Institute of Nuclear Physics (INFN), Largo B. Pontecorvo, 3 Pisa Italy; ^8^ Fondazione Pisana per la Scienza ONLUS San Giuliano Terme Italy; ^9^ NEST – Scuola Normale Superiore Pisa Italy; ^10^ Radiation Oncology Unit Pisa University Hospital “Azienda Ospedaliero‐Universitaria, Pisana” Pisa Italy; ^11^ Unit of Medical Physics Pisa University Hospital “Azienda Ospedaliero‐Universitaria, Pisana” Pisa Italy; ^12^ Department of Translational Research and New Technologies in Medicine and Surgery University of Pisa Pisa Italy; ^13^ Department of Pharmacy, School of Medical and Pharmaceutical Sciences University of Genoa Genoa Italy; ^14^ Centro 3R – Centro Interuniversitario per la promozione dei principi delle 3R nella didattica e nella ricerca Pisa Italy

**Keywords:** alternative biomodels, chorioallantoic membrane model, FLASH radiotherapy, head and neck carcinoma, topoisomerase inhibition

## Abstract

Locally advanced head and neck carcinoma remains associated with high morbidity and long‐term survival below 50%. Treatment commonly relies on cisplatin‐based chemoradiotherapy, which is effective but frequently associated with significant acute and chronic systemic toxicities. Therefore, safer and more effective therapeutic strategies are urgently needed. FLASH radiotherapy (RT) has emerged as a promising irradiation modality because of its potential to reduce damage to healthy tissues while preserving antitumor efficacy. Here, we investigated the anticancer activity of the Topoisomerase (Topo)‐II inhibitor ARN‐24139, alone and combined with FLASHRT, in human papillomavirus‐negative SCC‐25 head and neck carcinoma biomodels. Antitumor activity was assessed in 2D cell cultures using viability, apoptosis, clonogenic, wound‐healing, and γH2AX assays, as well as in SCC‐25 3D spheroids and in chorioallantoic membrane (CAM) tumor models. ARN‐24139 induced dose‐dependent cytotoxicity in SCC‐25 cells, with IC_50_ values of 7.3 ± 0.8 µM at 48 h and 7.2 ± 0.5 µM at 72 h, while showing limited toxicity in healthy HBEpC cells. Sequential low‐dose FLASH‐RT followed by ARN‐24139 enhanced antitumor activity, reducing cell viability at 4 Gy after 8 days and decreasing tumor growth and Ki67 expression in CAM models. These proof‐of‐concept findings support further investigation in more clinically representative and mechanistically informative HNSCC models.

## Graphical abstract

We report a proof‐of‐concept combination of topoisomerase inhibition and FLASH radiotherapy showing enhanced antitumor activity in head and neck carcinoma biomodels, supporting further investigation of this strategy in more clinically representative models and treatment settings.

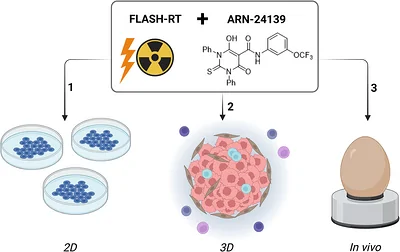

## 1 Introduction

Head and neck squamous cell carcinoma (HNSCC) comprises a heterogeneous group of epithelial malignancies arising in the upper aerodigestive tract, including the oral cavity, oropharynx, nasopharynx, hypopharynx, and larynx. Worldwide, approximately 660,000 new cases are diagnosed every year [1]. Traditionally, tobacco and alcohol consumption have represented the principal risk factors associated with HNSCC development; however, in recent decades, the incidence of HPV‐related tumors has markedly increased [2]. Although HPV‐positive patients generally exhibit improved prognosis and higher sensitivity to therapy compared with HPV‐negative cases, treatment regimens remain largely comparable between the two groups [2].

Therapeutic strategies for HNSCC depend on both tumor localization and disease stage. In patients with early‐stage oral tongue cancer, achieving optimal local disease control is essential for favorable long‐term outcomes [3]. Surgical resection, with or without postoperative RT, remains the standard approach for most T1–2N0 tumors depending on pathological risk factors, including positive surgical margins, vascular invasion, lymphatic invasion, and perineural infiltration. However, recent clinical guidelines do allow primary RT as a therapeutic option in early‐stage oral cavity cancers [4]. The use of primary RT has been recommended as an alternative for selected patients who are not considered for surgery due to old age, medical comorbidities, or concerns about functional or cosmetic outcomes [5, 6]. In locally advanced (LA) HNSCC (Stage III–IV), treatment generally relies on multimodal approaches that combine surgery, RT, and chemotherapy [7]. Concurrent chemoradiotherapy most commonly includes cisplatin administered every 3 weeks during RT at a dose of 100 mg m^−2^ body‐surface area [8]. Despite its clinical efficacy, cisplatin‐based therapy is frequently associated with severe systemic toxicities, including nephrotoxicity, ototoxicity, electrolyte imbalance, and myelosuppression, often requiring treatment modifications, particularly in elderly or fragile patients [8]. In addition, HPV‐negative tumors are characterized by aggressive clinical behavior and high recurrence rates, emphasizing the urgent need for safer and more effective therapeutic strategies [9].

Among alternative anticancer approaches, Topoisomerase (Topo)‐II inhibition represents a well‐established strategy because these enzymes play a critical role in resolving DNA topological stress during replication and are frequently overexpressed in malignant tissues [10, 11]. In particular, elevated Topo‐IIα expression in HNSCC has been associated with poor patient prognosis and reduced overall survival [12]. Etoposide, one of the most widely used Topo‐II inhibitors, has shown encouraging activity in clinical studies involving LA‐HNSCC patients. However, its clinical use remains limited by both acute toxicities and the risk of secondary malignancies, including therapy‐related leukemias [13].

Here, we have focused our attention on a novel class of Topo‐IIα inhibitors, which have been recently disclosed as hybrid compounds structurally inspired by the Food and Drug Administration‐approved etoposide anticancer drug and another anticancer Topo‐II inhibitor, called merbarone [14, 15]. ARN‐24139 was previously identified as one of the most promising compounds of this series because of its favorable drug‐like properties, in vivo pharmacokinetic profile, and cytotoxic activity against several tumor models, including prostate, cervical, and lung cancers [14]. Therefore, in this study, we have further investigated its antitumor potential against head and neck cancer, alone and combined with FLASH radiotherapy (FLASH‐RT)—an emerging technology that delivers ionizing radiations at ultra‐high dose rate (≥40 Gy/s) in milliseconds against minutes of conventional RT [16]. Increasing evidence suggests that FLASH‐RT can preserve normal tissues while maintaining antitumor efficacy, thereby improving the therapeutic ratio compared with conventional RT [17, 18]. Preclinical studies have already demonstrated the FLASH effect in several tumor models, including brain, breast, lung, ovarian, skin, and head and neck cancers [19, 20, 21]. Readers interested in the biological mechanisms underlying the FLASH effect may refer to the dedicated reviews on this topic [22].

In this study we evaluated the therapeutic efficacy of ARN‐24139 administered either alone or sequentially combined with FLASH‐RT in HPV‐negative SCC‐25 HNSCC models, including bi‐dimensional (2D) and three‐dimensional (3D) in vitro systems as well as alternative in vivo chorioallantoic membrane (CAM) models. ARN‐24139 exhibited a dose‐dependent anticancer effect in SCC‐25 cells while maintaining good tolerability in healthy cells, even at higher concentrations. We also evaluated the cell cycle regulation and DNA damage to verify the cytotoxicity of ARN‐24139. Enhanced viability reduction was recorded with the addition of FLASH‐RT, highlighting a synergistic effect between the two treatments. The tumor shrinkage observed in CAM models corroborated the synergy between ARN‐24139 and FLASH‐RT, which was further confirmed by the significant reduction of the proliferation marker (Ki67). Overall, our findings provide proof‐of‐concept evidence supporting further investigation of this combination in additional HNSCC models and treatment schedules.

## 2 Results

### 2.1 ARN‐24139 Induces Dose‐Dependent Cytotoxicity and DNA Damage in HPV‐Negative HNSCC Cells

**FIGURE 1:**
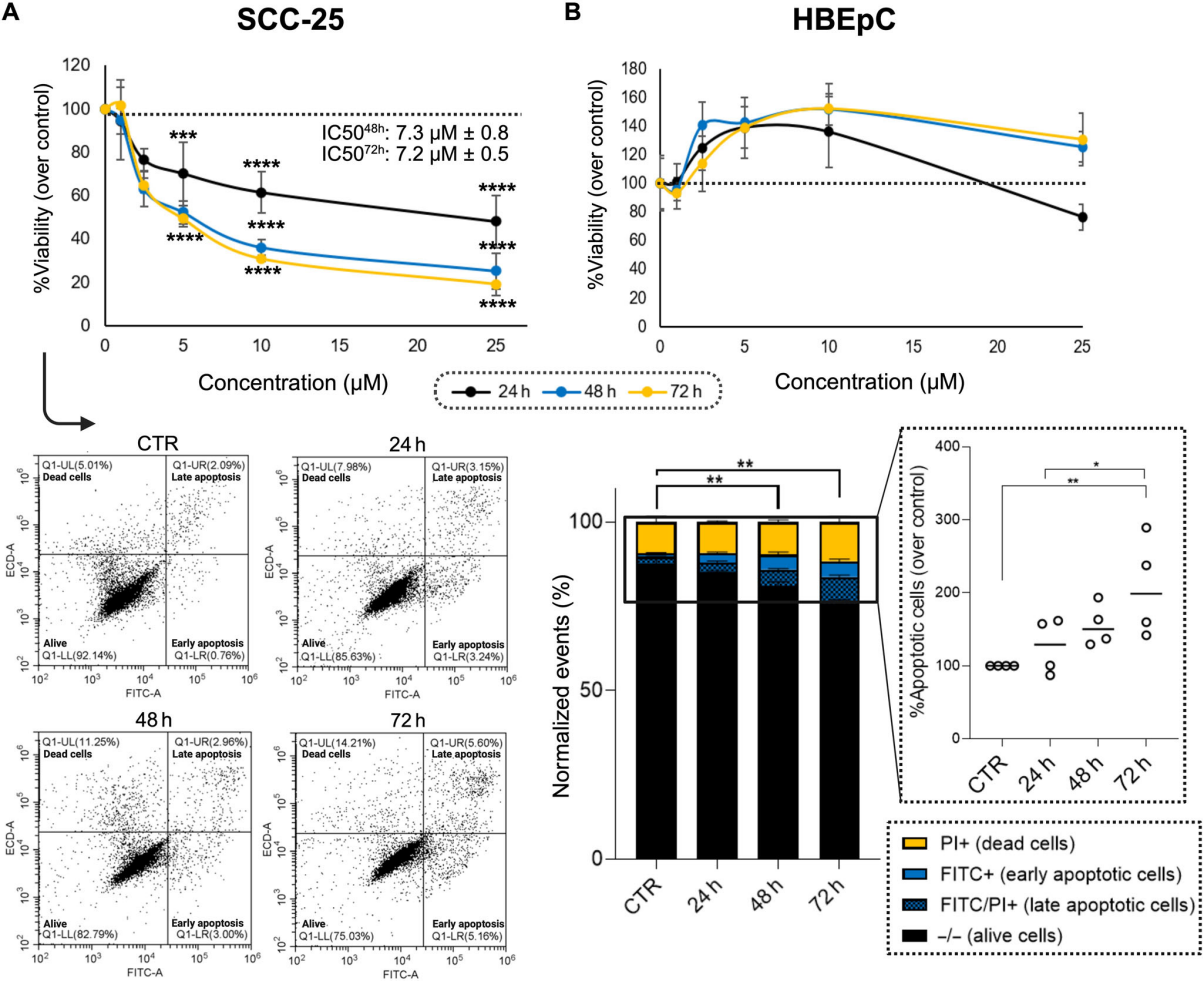
Antitumor effect of ARN‐24139 on SCC‐25 cells. (A, upper) SCC‐25 cells were treated at increased concentration of ARN‐24139 for 24 h and cell viability was measured up to 72 h. Data were normalized over the viability of control treated with medium. Data are reported as mean ± SD of three biological experiments, each with three experimental replicates. Statistical differences were calculated with two‐way ANOVA (Tukey's multiple comparison test; ****p < 0.0001. (A, bottom) Fluorescence‐activated cell sorting (FACS) has been performed by using the concentration of the IC_50_ value (∼7.3 µM) and the events of proliferation and apoptosis recorded up to 72 h. Data are first reported as % of normalized events of alive, late apoptotic, early apoptotic, and dead cells for each time point. The total events of apoptosis were normalized over the control, depicting a significant increase at 72 h (dashed box). Data are reported as mean ± SD of two biological experiments performed in duplicate. Statistical differences were calculated with unpaired *t*‐test. *Abbreviations*: PI: propidium iodide; FITC: fluorescein isothiocyanate. (B) HBEpC cells were treated with the same conditions of SCC‐25 cells and viability was measured up to 72 h. Data were normalized over the viability of control treated with medium. Data are reported as mean ± SD of three experimental replicates. Statistical analyses were performed using GraphPad Prism software.

To investigate the antitumor activity of the novel Topo‐II inhibitor ARN‐24139 against HPV‐negative HNSCC, SCC‐25 cells were initially treated with increasing drug concentrations and cell viability was monitored over time. Among the newly synthesized Topo‐II inhibitors previously developed by our group, ARN‐24139 exhibited the highest antiproliferative activity, with an IC_50_ of 7.3 ± 0.8 µM after 48 h and 7.2 ± 0.5 µM after 72 h, whereas the parent compounds etoposide and merbarone showed limited activity under the same experimental conditions (Figure 1A, upper panel and Figure S1A,B) [14]. The antiproliferative activity of others Topo‐II inhibitors of this chemical class against additional tumor cell lines is summarized in Table S1.

To further characterize the biological effects of ARN‐24139, apoptosis was evaluated by flow cytometry using the IC_50_ concentration. The fluorescence‐activated cell sorting (FACS) analysis demonstrated a progressive reduction in viable cells accompanied by a corresponding increase in both early and late apoptotic populations up to 72 h after treatment (Figure 1A, lower panel). The cytotoxicity of ARN‐24139 was also assessed in normal human bronchial epithelial cells (HBEpC), which maintained high viability throughout the experimental time course, showing only a slight transient reduction at the highest drug concentrations (Figure 1B). Under the same experimental conditions, cisplatin effectively reduced SCC‐25 viability but also significantly affected HBEpC cells (Figure S2).

**FIGURE 2:**
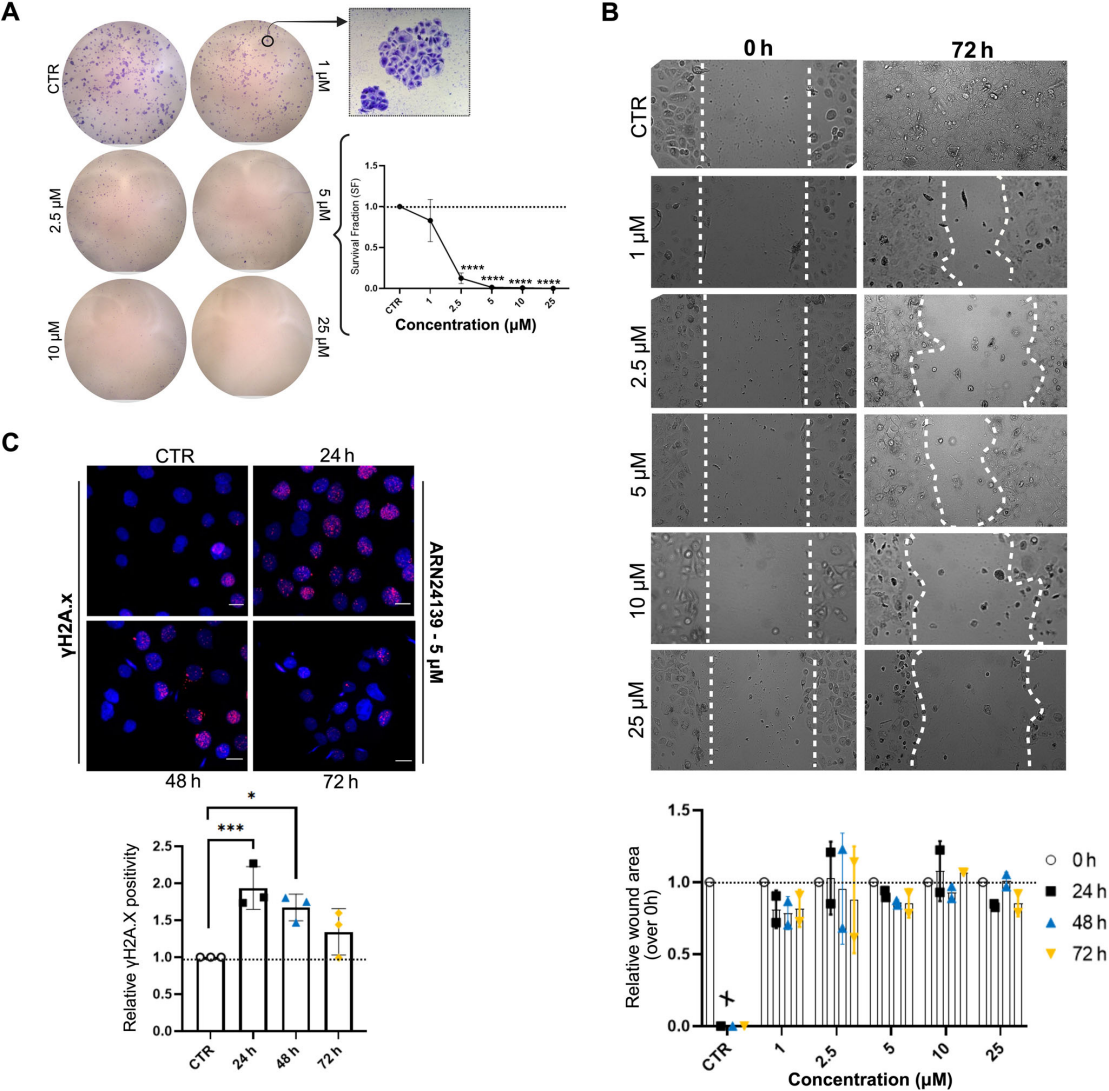
Validation of ARN‐24139 cytotoxicity in 2D models. (A) Clonogenic assay. Cells were seeded at different densities according to the concentration of the drug to allow the formation of colonies. A representative colony (∼50 cells) is depicted in the zoom image. Data are reported as mean ± SD of three biological experiments, each with two experimental replicates. Data are reported as % of survival fraction over the control. Statistical differences were calculated with two‐way ANOVA (Tukey's multiple comparison test; ****p < 0.0001. (B) Scratch assay. A small scratch was performed in the middle of the well with the p200 tip after removing the treatment. The wound closure was then monitored and images taken up to 72 h. ImageJ software was used to quantify the wound area by measuring the distance between the two edges of the scratch. Data are reported as mean ± SD of three biological experiments, each with two experimental replicates. Data are reported as a relative wound area over the time 0 h. (C) DNA damage detection. Cells were treated with 5 µM of ARN‐24139 for 24 h and double strand breaks were detected by using anti‐үH2A.x Ab and monitored up to 72 h (scale bar: 50 µm). Red dots indicate DNA damage foci. For each condition, at least five random fields were imaged. Data are reported as a relative DNA damage over the control and as mean ± SD of three biological experiments, each with three experimental replicates. Statistical differences were calculated with two‐way ANOVA (Tukey's multiple comparison test; ****p < 0.0001). Statistical analyses were performed using GraphPad Prism software.

The antitumor activity of ARN‐24139 was subsequently validated using complementary functional assays. Clonogenic assays demonstrated a dose‐dependent reduction in colony formation and survival fraction (SF) (Figure 2A). Similarly, wound‐healing experiments showed impaired migration of SCC‐25 cells following treatment, with incomplete wound closure observed even at the lowest tested concentration (Figure 2B). DNA damage analysis further confirmed the biological activity of ARN‐24139, revealing a significant increase in γH2AX‐positive foci 24 h after treatment, followed by a progressive decline at later time points (Figure 2C).

**FIGURE 3:**
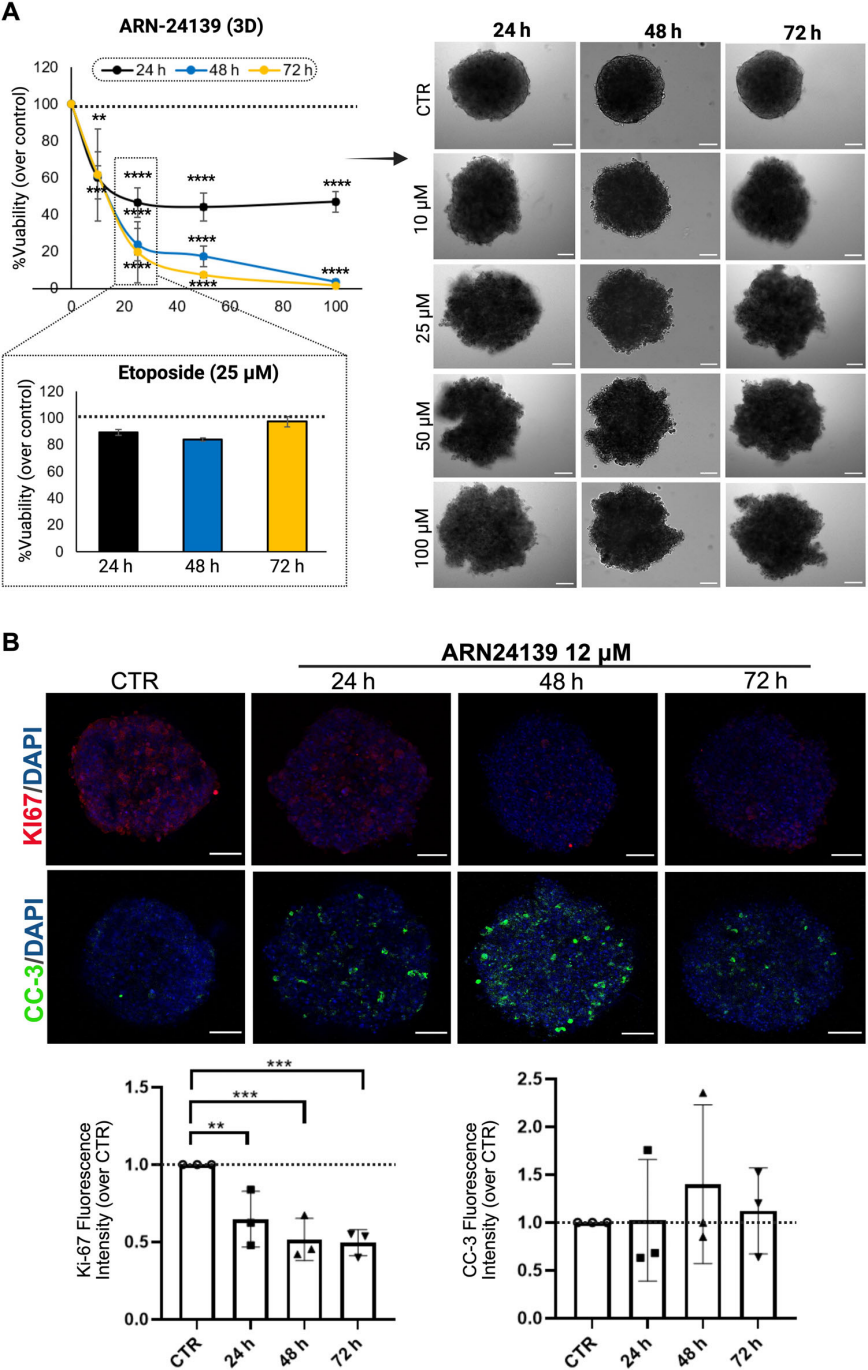
Cytotoxicity of ARN‐24139 on 3D tumor spheroids. (A, *left*) SCC‐25 3D cells were treated with 10× concentrated ARN‐24139 for 24 h and viability recorded up to 72 h. Etoposide was tested at single concentration (25 µM) for the three different time points. Data are reported as mean ± SD of three biological experiments, each with three experimental replicates. Data are reported as % viability over control. Statistical differences were calculated with two‐way ANOVA (Tukey's multiple comparison test; ****p < 0.0001. (A, *right*) SCC‐25 tumor spheroids were monitored and photographed at each time point. A significant structural damage and loss of sphericity was recorded for all the concentrations used (*scale bar: 100 µm*). (B) Evaluation of proliferation and apoptosis‐associated marker after treatment with ARN‐24139 (12 µM). For each spheroid, at least 50 µm in depth was covered with *z*‐stack acquisition and a Z project analysis was run on ImageJ software to sum positive signal. Red and green dots indicate signals for marker of proliferation (Ki67) and apoptosis cleaved Caspase‐3 (CC‐3) respectively. Blu signals are nuclei. Data are reported as mean ± SD of three biological experiments, each with at least 3 spheroids per condition. Data are reported as relative fluorescence intensity over control *(scale bar: 100 µm)*. Statistical differences were calculated with two‐way ANOVA (Tukey's multiple comparison test; ****p < 0.0001). Statistical analyses were performed using GraphPad Prism software.

To evaluate the activity of ARN‐24139 in a more physiologically relevant system, SCC‐25 tumor spheroids were generated and exposed to the compound [23]. Because of the increased complexity of the 3D model, ARN‐24139 was administered at concentrations 10‐fold higher than those used in 2D cultures. The compound maintained its antitumor activity, reaching an IC_50_ of 10.5 ± 3.1 µM after 48 h and 8.9 ± 4.8 µM after 72 h, whereas etoposide again showed limited efficacy (Figure 3A). Treatment induced a progressive loss of spheroid integrity and morphology in a dose‐dependent manner. Finally, immunofluorescence analysis demonstrated a significant reduction in Ki67 expression after treatment, whereas cleaved Caspase‐3 (CC‐3) showed only a modest, nonsignificant increase at 48 h (Figure 3B).

Overall, these findings demonstrate that ARN‐24139 exerts a potent antitumor effect against HPV‐negative HNSCC models, reducing cell viability, clonogenic potential, migration, and proliferation while inducing DNA damage in both 2D and 3D experimental systems.

### 2.2 ARN‐24139 Synergizes With Low‐Dose FLASH‐RT in HPV‐Negative HNSCC Cells

To investigate whether the therapeutic interaction between ARN‐24139 and FLASH‐RT was influenced by treatment sequence, three different administration schedules were evaluated. SCC‐25 cells were treated with ARN‐24139 24 h before irradiation, concomitantly with FLASH‐RT, or 48 h after FLASH irradiation. Cell viability was monitored up to 8 days after treatment to identify the most effective treatment schedule.

**FIGURE 4:**
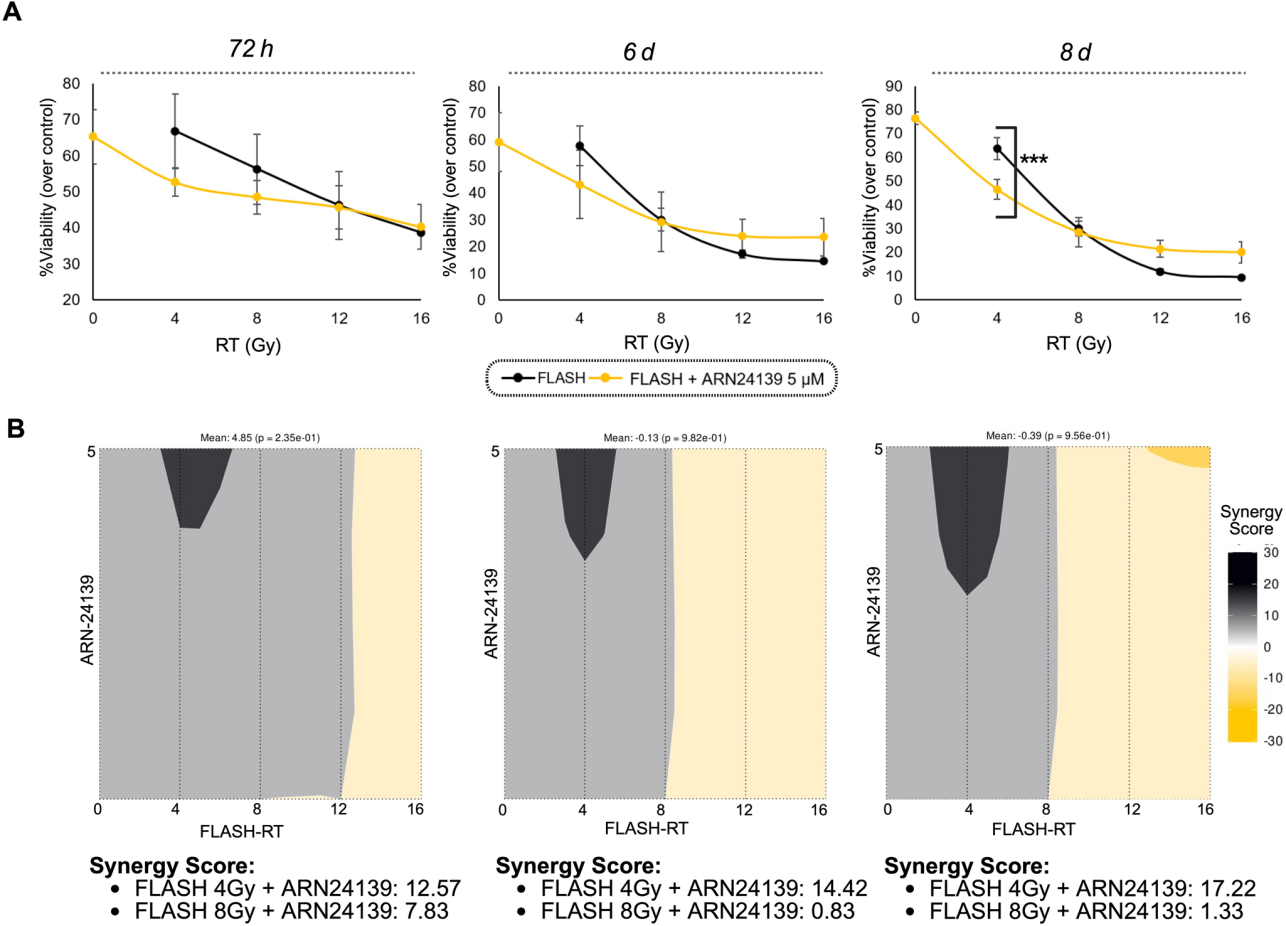
Evaluation of synergism between FLASH‐radiotherapy (RT) and ARN‐24139 in 2D models. (A) SCC‐25 cells were first irradiated at increased radiation dose (Gy) and after 48 h treated with ARN‐24139 (5 µM). Viability was recorded up to 8 days post‐irradiation. A significative difference between FLASH‐RT alone and combined approach was recorded at 8 days with low dose of radiation (4 Gy). Data are reported as mean ± SD of three biological experiments, each performed in triplicate. Statistical differences were calculated with two‐way ANOVA (Tukey's multiple comparison test; ****p < 0.0001). (B) Calculation of synergism performed by using a free online tool (synergyfinder.org). The degree of combination synergy, or antagonism, is analyzed using SynergyFinder. The reported synergy score was calculated using the Highest Single Agent (HAS) reference model, which compares the observed combination response with the expected response under a no‐interaction assumption. The red portion of the panels indicate synergism, while the green portion indicates antagonism. A higher synergy score was recorded at 8 days post‐treatment in accordance with the viability test. Statistical analyses were performed using GraphPad Prism software.

Among the tested treatment schedules, administration of ARN‐24139 48 h after FLASH irradiation produced the greatest reduction in cell viability compared with FLASH‐RT alone, particularly at the lowest radiation dose (4 Gy) (Figure 4). No significant improvement was observed when ARN‐24139 was administered before or concomitantly with FLASH irradiation (Figure S3). Based on these findings, the 48 h interval was selected for all subsequent combination experiments. Using this treatment schedule, the antitumor efficacy of FLASH‐RT combined with ARN‐24139 was further evaluated over time. A progressive reduction in cell viability was observed in the combination group at all evaluated time points following 4 Gy irradiation. Although this trend did not reach statistical significance at 72 h and 6 days, a significant decrease in cell viability compared with FLASH‐RT alone was observed after 8 days (Figure 4A). Synergy analysis confirmed a synergistic interaction between FLASH‐RT and ARN‐24139 at 4 Gy at all evaluated time points, with the highest synergy score recorded at 8 days post‐treatment. Only limited synergism was detected at higher radiation doses (8 Gy), whereas no synergistic interaction was observed at doses ≥12 Gy (Figure 4B). Unsurprisingly, the trend was lost in the synergistic evaluations on 3D models (Figure S4).

To determine whether this interaction was specific to FLASH irradiation, SCC‐25 cells were also treated with conventional radiotherapy (CONV‐RT) followed by ARN‐24139 using the same treatment schedule. Similar to FLASH‐RT, the combination significantly reduced cell viability compared with CONV‐RT alone at 4 Gy after 8 days, whereas no additional benefit was detected at higher radiation doses. Synergy analysis similarly identified the strongest interaction at the lowest radiation dose (Figure S5).

Overall, these results demonstrate that ARN‐24139 enhances the antitumor activity of both FLASH‐RT and CONV‐RT under optimized treatment conditions, with the greatest synergistic effect observed at low radiation doses.

### 2.3 Combined FLASH‐RT and ARN‐24139 Treatment Reduces Tumor Growth in CAM Models

**FIGURE 5:**
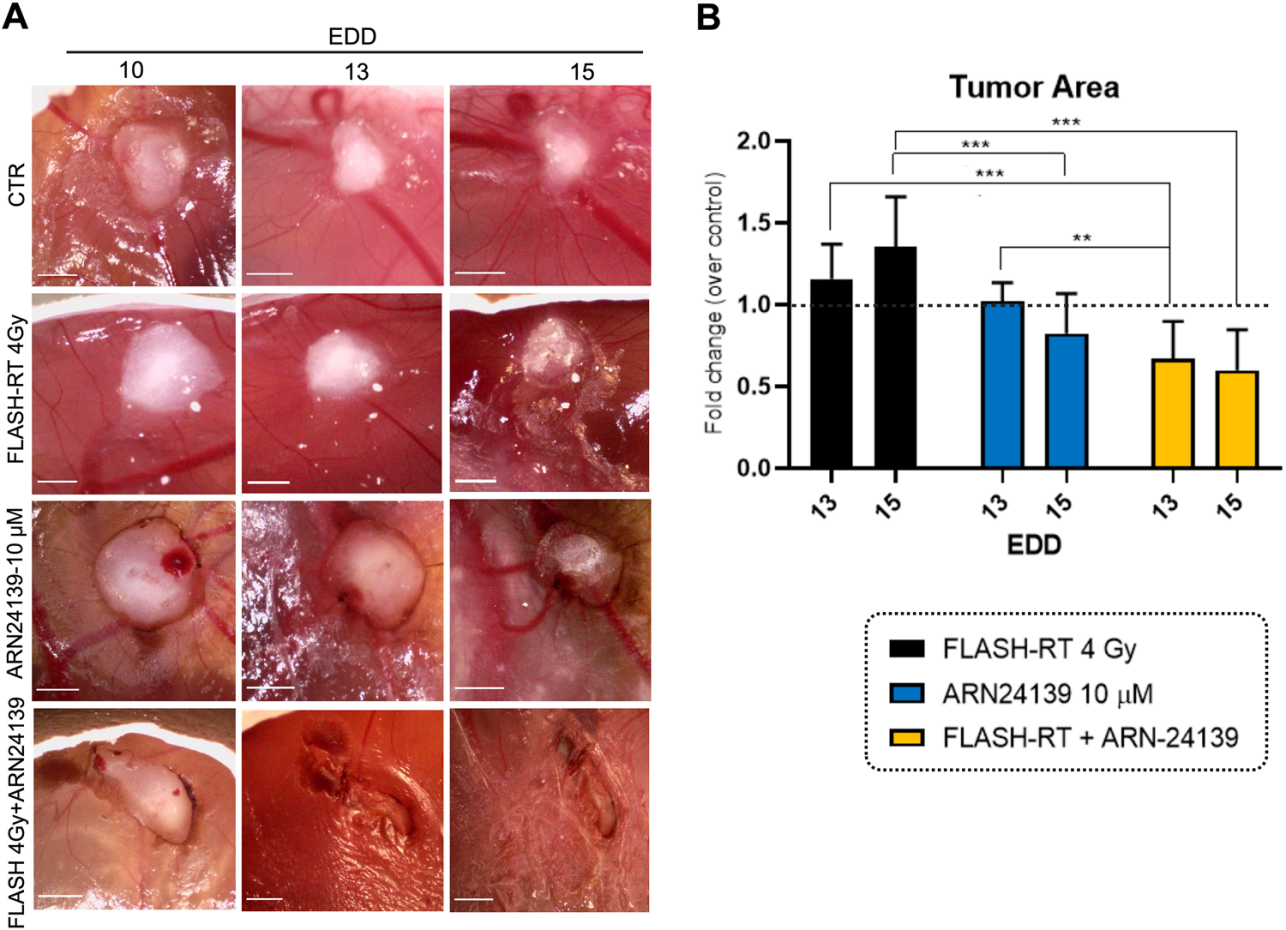
Chorioallantoic membrane (CAM) assay. (A) Representative images of SCC‐25 tumors grown on CAM. Xenograft tumors were photographed with DinoLite microscope and monitored until embryonic day of development (EDD) 15 (*scale bar: 2 mm*). Tumor shrinkage is significantly visible at Day 15 for the monomodal conditions, and immediately from Day 13 for the combined administration. (B) Area of xenograft tumors was determined by drawing a precise ROI around the tumors with free hand selection in Image J software and the mean values were normalized over the control (set as 1, dashed line). In the combined approach, tumor‐bearing embryos were first irradiated at EDD10 with FLASH 4 Gy and then treated with ARN‐24139 (10 µM) at EDD12 (in accordance with in vitro experiments). For the monomodal conditions, eggs were either irradiated at EDD10 or treated with the drug at EDD12 respectively. Data represent the pooled results of three independent biological experiments, with a total of 15 embryos analyzed per treatment condition. Data are reported as fold change over control. Statistical differences were calculated with two‐way ANOVA (Tukey's multiple comparison test; ****p < 0.0001). Statistical analyses were performed using GraphPad Prism software.

The antitumor activity of ARN‐24139 as a single and combined approach was further investigated by following our optimized procedures using the alternative in vivo CAM model of HNSCCs [24, 25]. The same treatment design was maintained across both in vitro and in vivo systems to ensure consistency among experimental models. Based on the previous findings, the concentration of the IC_50_ for ARN‐24139 obtained on 3D models (10 µM) and the lowest dose of RT (4 Gy) that promoted the synergistic effect were employed. After randomization of tumor‐bearing embryos, four treatment conditions were assessed: (i) control, (ii), FLASH‐RT 4 Gy, (iii) ARN‐24139 10 µM, (iv) FLASH‐RT 4 Gy + ARN‐24139 10 µM (Figure 5).

FLASH‐RT was delivered at embryonic day of development (EDD) 10 and the inhibitor at EDD12 in order to be consistent with the 48 h interval between the two administrations of the in vitro experiments. Tumors were photographed and monitored up to Day 15 and their area was determined.

FLASH‐RT alone did not significantly affect tumor growth compared with untreated controls, whereas ARN‐24139 alone induced a measurable reduction in tumor size. The combination treatment produced the greatest antitumor response, resulting in a significant decrease in tumor area compared with both the control and monotherapy groups (Figure 5A,B).

**FIGURE 6:**
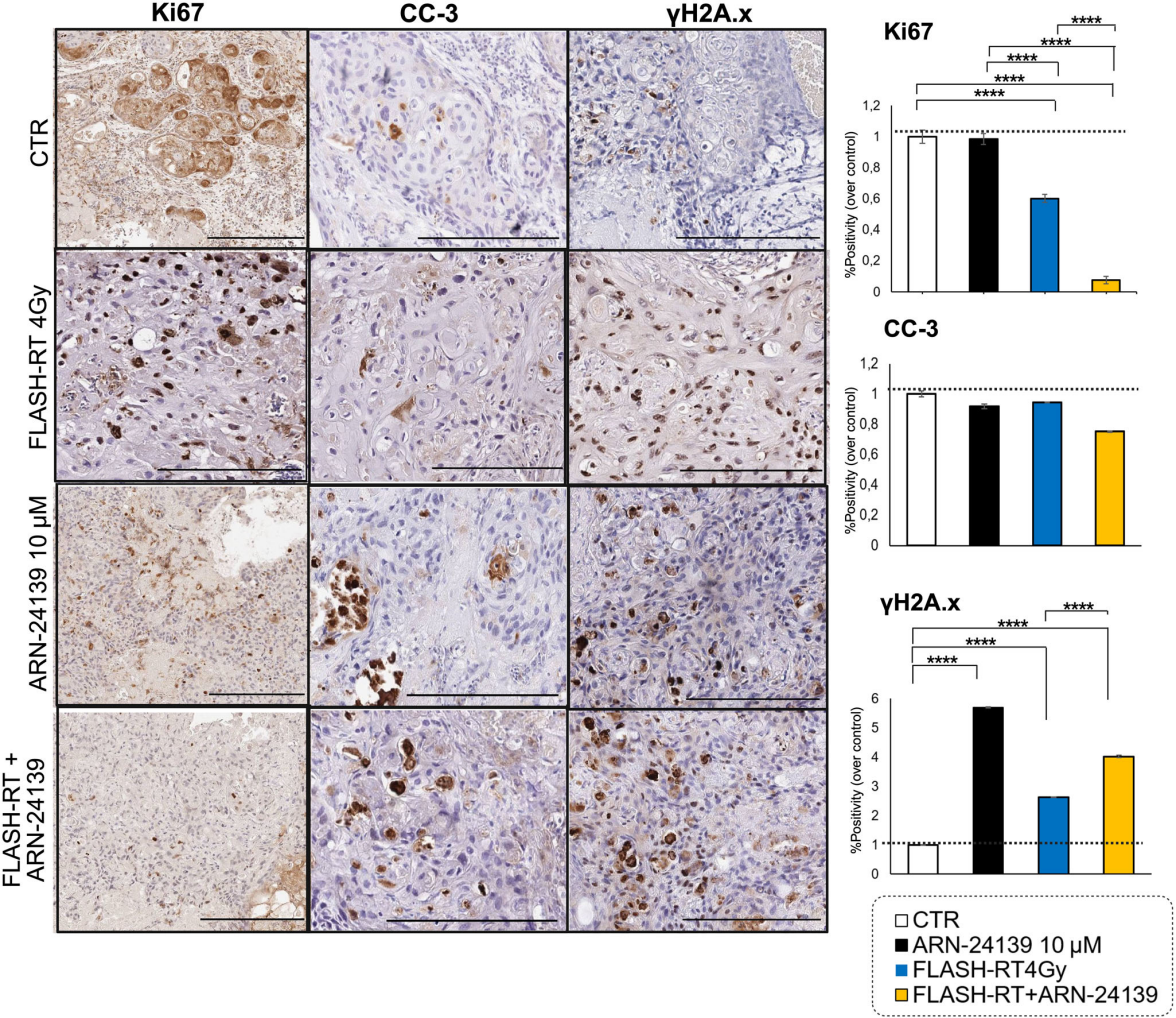
Immunohistochemistry (IHC) on CAM samples. At EDD15, tumors were harvested and fixed with formalin for 48 h. Subsequently, tumors were processed by IHC and the expression of the proliferation marker (Ki67), cleaved Caspase‐3 (CC‐3), and DNA damage (γH2A.x) evaluated. The brown signal indicates the respective protein expression (*scale bar: 200 µm*). The quantitative assessment was performed with ImageScope software by using a specific algorithm (Positive Pixel Count 2004‐08‐11). At least five random areas of the slide section were quantified. Data are reported as % of positivity over control. Statistical differences were calculated with two‐way ANOVA (Tukey's multiple comparison test; ****p < 0.0001). Statistical analyses were performed using GraphPad Prism software.

To further characterize the biological response to treatment, tumors were collected at EDD15 and analyzed by immunohistochemistry (IHC) to assess proliferation, apoptosis, and DNA damage. Quantification of Ki67 expression revealed a significant reduction in proliferative activity following the combined approach compared with ARN‐24139 alone (Figure 6). In contrast, lack of changes was reported for CC‐3 expression whereas γH2AX staining revealed increase in DNA damage in treated tumors, although no clear additive effect was observed between the individual treatments and the combination (Figure 6).

Overall, these findings demonstrate that sequential administration of FLASH‐RT followed by ARN‐24139 effectively suppresses tumor growth in CAM xenografts and is associated with reduced tumor cell proliferation together with increased DNA damage.

## 3 Discussion

HNSCC remains a challenging malignancy because current treatment strategies rely heavily on cisplatin‐based chemoradiotherapy, which is frequently associated with severe acute and long‐term toxicities that negatively affect patients' quality of life [1]. In this context, identifying novel therapeutic approaches capable of maintaining antitumor efficacy while improving treatment tolerability represents an important unmet clinical need. Topo‐IIα has emerged as a promising therapeutic target in HNSCC, as its overexpression has been associated with aggressive tumor behavior and poor clinical outcome (Figure S6) [11, 12]. Building upon our previous work describing the synthesis and biological characterization of ARN‐24139, the present study provides the first proof‐of‐concept evaluation of its combination with FLASH‐RT, exploring whether Topo‐II inhibition may represent a suitable strategy to enhance radiation response in HNSCC.

ARN‐24139 demonstrated potent antitumor activity across both 2D and 3D experimental models, reducing cell viability, clonogenic survival, migration, and proliferative capacity while increasing DNA damage. Importantly, unlike cisplatin, ARN‐24139 showed limited cytotoxicity toward normal epithelial cells under the same experimental conditions. Although direct comparisons between the two compounds should be interpreted with caution, these preliminary findings suggest that selective Topo‐II inhibition may provide a more favorable therapeutic window than conventional platinum‐based chemotherapy. The preservation of antitumor activity in 3D spheroids indicates that the efficacy of ARN‐24139 is maintained even in more physiologically relevant tumor models characterized by increased structural complexity and reduced drug penetration, supporting its further preclinical development [9, 26].

The main goals of combined administrations are to reduce both off‐target toxicity by minimizing doses and improve outcomes by escalating effects [27]. By definition, a synergistic effect occurs when two or more treatments promote a therapeutic response greater than the sum of their individual effects [28]. In the present study, the efficacy of the combination was highly dependent on treatment sequence. Although the treatment schedule adopted here differs from conventional clinical protocols for LA‐HNSCC, the present work was designed as a proof‐of‐concept preclinical investigation aimed at evaluating the biological interaction between ARN‐24139 and FLASH‐RT rather than reproducing current therapeutic regimens. The 48 h interval between irradiation and drug administration was selected based on the experimental observation that this sequence generated the most pronounced antitumor effect among the tested conditions. However, this regimen does not reproduce standard concurrent chemoradiotherapy used in LA‐HNSCC. Therefore, the clinical feasibility of this schedule remains uncertain. Future work should evaluate fractionated irradiation protocols and clinically relevant timing regimens to determine whether the observed interaction can be translated into treatment schedules compatible with current oncological practice.

Although the molecular basis underlying this temporal dependency was not directly investigated, delayed drug administration may allow the establishment of radiation‐induced cellular responses, including DNA damage accumulation and alterations in cellular susceptibility to subsequent treatments [29]. Future mechanistic studies will therefore be necessary to determine whether DNA damage response pathways, DNA repair dynamics, or radiation‐induced changes in cell‐cycle progression contribute to the observed treatment interaction.

Interestingly, the synergistic response was restricted to low radiation doses, whereas antagonistic interactions emerged at higher FLASH doses. While this finding initially appeared counterintuitive, increasing evidence indicates that extensive radiation‐induced DNA damage activates cell‐cycle checkpoints, potentially leading to prolonged cell‐cycle arrest and alterations in cellular proliferation dynamics [30, 31]. Since Topo‐II inhibitors primarily target actively proliferating cells, radiation‐induced changes in cell‐cycle progression may reduce cellular susceptibility to subsequent Topo‐II inhibition. Furthermore, activation of DNA repair pathways following extensive radiation‐induced damage may also influence treatment responsiveness [32]. Although these mechanisms remain speculative, acknowledging these possibilities provides a biologically plausible framework for interpreting the observed dose‐dependent interaction and highlights the need for dedicated mechanistic investigations. Moreover, the expected loss of this trend observed in 3D models may be attributed to the transient expansion of the hypoxic core that engulfs a large number of well‐oxygenated cells during FLASH‐RT making the spheroids radioresistant, with respect to the conventional RT in which oxygen is steadily replenished [33].

An additional finding of the present study is the comparable synergistic interaction observed when ARN‐24139 was combined with CONV‐RT using the same treatment schedule. This comparison allowed us to distinguish the radiosensitizing effect of ARN‐24139, which was observed under both irradiation modalities, from the broader therapeutic rationale for FLASH‐RT, which is mainly related to its reported ability to improve the therapeutic ratio through normal tissue sparing. This observation suggests that the enhanced antitumor response primarily derives from the interaction between Topo‐II inhibition and radiation‐induced DNA damage rather than from FLASH‐specific biological mechanisms. Nevertheless, this result should not be interpreted as diminishing the therapeutic value of FLASH irradiation. Accumulating preclinical evidence indicates that the principal advantage of FLASH‐RT resides in its ability to preserve normal tissues while maintaining tumor control comparable to CONV‐RT [16, 22]. Consistent with these observations, we previously demonstrated in CAM tumor models that FLASH irradiation improves the therapeutic ratio by reducing radiation‐induced toxicity without compromising antitumor efficacy [18]. Therefore, although normal tissue sparing effect was beyond the scope of the present study, combining ARN‐24139 with FLASH‐RT may still represent a promising strategy for further improving the therapeutic index of radiation treatment.

The CAM model provided additional support for the biological activity of the optimized treatment schedule, confirming significant tumor growth inhibition together with reduced Ki67 expression following combination treatment. Since Ki67 is a well‐established marker of tumor proliferation and an important prognostic indicator in several malignancies [34], these findings reinforce the antiproliferative activity of ARN‐24139 observed in vitro. In contrast, the lack of significant changes in CC‐3 expression suggests that apoptosis alone may not fully explain the observed antitumor response, which would partially confirm the paradoxical role of the caspase cascade in ionizing radiation therapy [35, 36]. This apparent discrepancy suggests that tumor regression may not be exclusively driven by apoptosis or may involve cell death mechanisms not fully captured by the selected marker. Since CC‐3 reflects only one component of apoptotic signaling, additional pathways such as cell‐cycle arrest, mitotic pertubation, alternative caspase activation, or non‐apoptotic cell death mechanisms may also contribute to the observed antitumor effects. Consequently, the present findings support the hypothesis that multiple complementary mechanisms may contribute to the antitumor efficacy of the combined administration.

Although the present findings provide encouraging evidence supporting the therapeutic potential of ARN‐24139 combined with FLASH‐RT, several limitations should be acknowledged. This study was conducted using a single HPV‐negative HNSCC cell line and therefore may not fully capture the biological heterogeneity of head and neck tumors. In addition, cisplatin‐based chemoradiotherapy represents the current standard of care for LA HNSCC, and the present work did not include a direct comparison between ARN‐24139 combined with FLASH‐RT and conventional chemoradiotherapy (cisplatin plus CONV‐RT). Such comparisons will be essential to better define the potential advantages of this novel therapeutic strategy. Since ARN‐24139 represents a new Topo‐II inhibitor with a distinct pharmacological profile, the experimental design did not reproduce conventional fractionated clinical treatment schedules, and the CAM model lacks a mature adaptive immune system, preventing the evaluation of immune‐mediated effects potentially associated with FLASH‐RT. Moreover, immunogenic cell death‐related markers, including calreticulin exposure, HMGB1 release, and ATP release, were not evaluated in the present study. Therefore, potential FLASH‐specific immunological effects remain outside the scope of this work and should be investigated in future studies using immunocompetent mammalian models. Although the CAM assay provided an important intermediate in vivo validation, confirmation in advanced mammalian models will be necessary to assess long‐term therapeutic efficacy, systemic safety, and translational applicability. Finally, the molecular mechanisms underlying some observed responses, including the treatment sequence dependency and the antagonistic interaction observed at higher radiation doses, remain incompletely characterized. Consequently, the present work should be considered an early‐stage preclinical proof‐of‐concept investigation. Future studies involving multiple HPV‐positive and HPV‐negative HNSCC models, including cell lines with distinct anatomical origins, molecular features, and radiosensitivity profiles, together with direct comparison with standard chemoradiotherapy, validation in mammalian in vivo systems, and dedicated mechanistic investigations, will be required to fully define the translational potential of this therapeutic strategy.

## 4 Conclusions

The present study demonstrates the therapeutic potential of the novel Topo‐II inhibitor ARN‐24139 in HPV‐negative HNSCC experimental models. Across both 2D and 3D in vitro systems, ARN‐24139 induced a clear dose‐dependent cytotoxic response associated with increased DNA damage, reduced proliferation, and apoptosis induction. These findings further support the relevance of Topo‐IIα inhibition as a promising strategy against aggressive HNSCC tumors, particularly considering the poor prognosis and limited therapeutic options currently available for HPV‐negative disease.

An important aspect of this work is the investigation of ARN‐24139 in combination with FLASH‐RT. While conventional chemoradiotherapy remains the standard treatment for LA HNSCC, its clinical use is often limited by severe systemic toxicities associated with platinum‐based regimens. In this context, the observed synergistic effect between FLASH‐RT and ARN‐24139 under sequential administration conditions is particularly encouraging. Notably, the enhanced antitumor activity obtained with low radiation doses may support future strategies aimed at maximizing therapeutic efficacy while minimizing treatment‐related toxicity.

The results obtained in CAM tumor models provide an additional level of preclinical support for this combinatorial approach. In particular, the significant reduction in tumor growth and Ki67 expression supports the biological effectiveness of combining FLASH‐RT with Topo‐II inhibition in vivo. At the same time, the differences observed between 2D and 3D systems underline the importance of employing experimental models with increasing biological complexity to better reproduce tumor microenvironment dynamics and treatment resistance mechanisms.

## 5 Materials and Methods

### 5.1 Chemistry

Compound ARN‐24139 was synthesized as previously reported by Arencibia et al. [14]. Synthesis procedures and nuclear magnetic resonance of others Topo‐II inhibitors and decatenation assay are reported in Supporting Information: Chemistry section.

### 5.2 Cell Culture (2D and 3D)

Human squamous cell carcinoma SCC‐25 cells (RRIDs: CVCL_1682) and HBEpC (ATCC PCS‐300‐010) were obtained from the American Type Culture Collection (ATCC) and routinely tested to confirm the absence of mycoplasma contamination. SCC‐25 cells were cultured in a 1:1 mixture of Dulbecco's modified Eagle medium (DMEM) and Ham's F12 medium supplemented with 10% fetal bovine serum (FBS), 4 mM l‐glutamine, 1 mM sodium pyruvate, 100 U/mL penicillin, 100 mg/mL streptomycin (Invitrogen), and 400 ng/mL hydrocortisone. Cells were maintained at 37°C under a humidified atmosphere containing 5% CO_2_. HBEpC cells were instead cultured in DMEM supplemented with 10% FBS, 4 mM L‐glutamine, 1 mM sodium pyruvate, and antibiotics. For 3D cultures, SCC‐25 spheroids were generated using ultra‐low attachment 96‐well plates (Costar Ref:7077). Briefly, cells were detached, counted, and seeded at a density of 10,000 cells per well. Plates were then incubated for 24 h to allow spheroid formation.

### 5.3 Viability Assay on 2D Cultures

The cytotoxicity of ARN‐24139 was evaluated using the WST‐8 colorimetric assay based on the tetrazolium salt 2‐(2‐methoxy‐4‐nitrophenyl)‐3‐(4‐nitrophenyl)‐5‐(2,4‐disulfophenyl)‐2H‐tetrazolium monosodium salt. SCC‐25 and HBEpC cells were seeded into 96‐well plates and allowed to adhere overnight before treatment with increasing concentrations of ARN‐24139 for 24 h at 37°C. For each experimental time point cells were incubated with 10% WST‐8 reagent (10 µL) and 10% serum‐containing medium (90 µL) for 2 h. Absorbance (450 nm) was subsequently measured using a microplate reader (Glomax Discovery; Promega, Madison, WI, USA). Cell viability was expressed relative to untreated control cells, which were considered as 100% viable. Data are presented as the mean ± SD of three independent experiments, each performed with three technical replicates. The viability assay of the other cancer cell lines is reported in Supporting Information: Biology section.

### 5.4 Cell Culture Condition and Viability Assay of Additional Tumor Cell Lines

Human cancer cell lines A549 (lung adenocarcinoma, ATCC CCL‐185, RRIDs: CVCL_0023), DU‐145 (androgen‐independent prostate cancer, ATCC HTB‐81, RRIDs: CVCL_0105), and HeLa (cervical carcinoma, ATCC CCL‐2, RRIDs: CVCL_0030) were purchased from ATCC and mycoplasma‐free. Hela and DU145 cells were routinely grown in minimal essential medium containing Eagle's salts and l‐glutamine supplemented with 10% heat‐inactivated FBS; A549 cells were grown in F12K (Gibco) supplemented with 2 mM l‐glutammine, 10 % FBS, all the cell lines were grown in a humidified atmosphere of 5% CO2 at 37°C. To assess the antiproliferative activity of the compounds, cells were seeded at a density of 2500 cells/well (HeLa) or 5000 cells/well (A549, DU‐145) in 96‐well plates, and cell viability was measured using the MTT assay as described previously [1]. Values are reported as the mean ± SD of two independent experiments.

### 5.5 Viability Assay on 3D Models

Cell viability in 3D spheroids was evaluated using the CellTiter‐Glo 3D Cell Viability Assay (Promega, Milan, Italy). Tumor spheroids were exposed to increasing concentrations of ARN‐24139, administered at 10‐fold higher concentrations compared with 2D cultures to account for the increased resistance and reduced penetration associated with 3D systems. After 24 h of treatment at 37°C, spheroids were transferred into white 96‐well plates containing 100 µL of medium for luminescence measurements. An equal volume of CellTiter‐Glo 3D reagent was then added to each well. Plates were shaken for 5 min and incubated for an additional 25 min before signal acquisition using a GloMax Discovery microplate reader (Promega). Viability values were normalized to untreated spheroids. Data are reported as mean ± SD of three independent experiments performed in triplicate. Spheroid morphology was monitored and imaged at each time point using a Leica DMI400B microscope.

### 5.6 Clonogenic Assay

For clonogenic assays, SCC‐25 cells were seeded into six‐well plates at different densities according to the drug concentration tested. After overnight incubation, cells were treated with increasing concentrations of ARN‐24139 for 24 h. Following treatment removal, cells were washed and cultured for an additional week to allow colony formation. Colonies were subsequently fixed and stained using a 6% crystal violet solution (Thermo Scientific; Cat: 405830250) prepared in ethanol. Only colonies containing more than 50 cells were included in the analysis. Colony counting was performed using ImageJ software. The survival fraction (SF) was calculated as the ratio between the plating efficiency (PE) of treated cells and that of untreated controls. PE was determined by dividing the number of colonies formed by the number of seeded cells. Data are expressed as mean ± SD of three independent experiments performed in duplicate.

### 5.7 Wound Healing Assay

Cells were seeded in 12‐well plates at a density of 12 × 10^3^ cell/well and the day after they were treated with increased concentrations of ARN‐24139 for 24 h. Treatment was then removed, and a small scratch was performed with a p200 tip. The wound closure was monitored and images taken at each time point with a Leica microscope DMI400B. The data were normalized and reported as fold change of relative wound area over control. Data are reported as mean ± SD of two independent experiments performed in duplicate.

### 5.8 Immunocytochemistry on 2D Models

SCC‐25 cells were seeded in WillCo‐dish Glass Bottom dishes at a concentration of 8 × 10^5^ cells/well and then placed in the incubator to let them attach for 24 h. Upon reaching about 70–80% confluence, the cells were treated with ARN‐24139 (5 µM) for 24 h. After treatment, cells were fixed with 4% paraformaldehyde (PFA) for 20 min. After aspirating PFA, the cells were washed three times with phosphate‐buffered saline (PBS; 1×) and permeabilized with 0.1% Triton‐PBS for 10 min at room temperature (RT). Cells were then incubated with blocking solution (5% bovine serum albumin (BSA) in 1× PBS) for 1 h to avoid unspecific signals. Cells can stay in the blocking solution for few weeks if stored at 4°C. Cells were then incubated with anti Phospho‐Histone H2A.X (Ser139) antibody (Cell Signaling #9718; Rabbit mAb) diluted 1:400 in 1% BSA in PBS overnight at 4° in a humid chamber. Then, the cells were washed thrice with PBS and incubated in dark with the secondary antibody conjugated with AlexaFluor‐647 (diluted 1:150 in 1.5% BSA in PBS) for 1.5 to 2 h at RT. After two washes with PBS, the cells were incubated with DAPI (diluted 1:500 in PBS1×) for 15 min at RT and then washed twice in PBS.

### 5.9 Immunocytochemistry on 3D Models

The spheroids were transferred to 2 mL Eppendorf and then treated with ARN‐24139 (12 µM) for 24 h. After treatment spheroids were washed twice in PBS1X and then fixed with 4% PFA for 20 min at RT. After several washes, spheroids were permeabilized in a solution of 0.1% Triton x‐100 in PBS1X for 4 min. After permeabilization, a blocking solution (5% BSA in 1× PBS) was then added to the spheroids for 1 h at RT. Blocking solution was removed and the primary antibody (anti‐Ki67 monoclonal Ab; Invitrogen MA5‐15690, diluted 1:300 or anti‐CC‐3 (Asp175) Rabbit, diluted 1:400; Cell Signaling #9661) added in a solution of 1.5% BSA in PBS1X overnight at 4°. The day after samples were washed three times in PBS1× and incubated for 1.5 h with secondary antibody at RT (anti‐mouse AlexaFluor‐488 diluted 1:200 for Ki67, and goat anti‐rabbit AlexaFLuor488 diluted 1:200 for CC‐3).

### 5.10 Confocal Microscope

Imaging of ICC experiments was performed by using a Leica TCS SP5 SMD inverted confocal microscope (Leica Microsystems AG), equipped with an argon laser (488 nm excitation) and an external laser (405 nm excitation). A 40× 1.5 NA oil immersion objective (Leica Microsystems) was used for visualization. For each condition, at least five random fields were imaged. Nuclei and γH2AX foci were counted, and foci numbers were normalized over the number of nuclei. Fold changes were calculated relative to control samples. Error bars indicate the standard deviation (SD) of three independent experiments. Image analysis was performed by using ImageJ software. For 3D models, at least three random spheroids were imaged. A z‐stack was acquired each time, covering at least 50 µm in depth. For the analysis, a Z project was run on ImageJ and the background was removed by drawing a region of interest (ROI) outside the spheroid. Then, the free hand selection was used to draw the whole area of the spheroid and the mean intensity calculated. Data were normalized with respect to the control and reported as fold change value. Error bars indicate the SD of three independent experiments.

### 5.11 Fluorescence‐Activated Cell Sorting

Cells were treated with ARN‐24139 for 24 h, washed twice with PBS, and subsequently cultured in complete medium. Apoptosis was evaluated at 24, 48, and 72 h post‐treatment. At each time point, cells were trypsinized, harvested, and stained using the Annexin V‐FITC Apoptosis Detection Kit (eBioscience, Invitrogen, Waltham), according to the manufacturer's instructions. Stained cells were immediately analyzed by flow cytometry (CytoFLEX S3; Beckman Coulter). Data were collected from three independent experiments and used for statistical analysis.

### 5.12 In Vitro Irradiation

All irradiations were carried out at the Centro Pisano FLASH Radiotherapy (CPFR) using a dedicated linear accelerator, ElectronFlash, specifically designed for FLASH experiments [37]. This linac delivers electron beams at 7 or 9 MeV and provides extensive control over beam parameters: the electron beam current can be set between 1 and 100 mA, pulse durations range from 0.5 to 4 µs, and the pulse repetition frequency (PRF) can be adjusted from 1 to 245 Hz without affecting the beam's energy spectrum. Such versatility allows independent adjustment of both the average dose rate (ADR) and the dose per pulse and switch between conventional (CONV) and FLASH irradiation. A detailed overview of the irradiation parameters used in this study is presented in Table S2. For all in vitro experiments, both CONV and FLASH irradiations were performed using the Ø100 mm applicator. This setup provided a highly uniform dose distribution, maintaining over 95% homogeneity across the entire cell irradiation area, thus allowing a minimum spatial dose variability. The linear accelerator operated in a vertical configuration, with Petri dishes placed on a 12 mm solid water slab, corresponding to the build‐up depth for the selected energy (Figure S7). In these experiments the PRF was changed to maintain an average dose rate of 240 Gy/s, a value that exceeds the commonly reported threshold needed to trigger the FLASH effect (100 Gy/s), across all the dose values used in this study. This value can also be maintained constant at each total dose value chosen for this study. The ADR is defined, neglecting the pulse size, as:

$$\text{DR}=\frac{n}{n-1}\times \text{DPP}\times \text{PRF}$$

The dosimetric characterization of our beams was performed using the flashDiamond (fD) detector, and other dosimeters and techniques [38, 39, 40, 41]. The beam output variations were considered using the beam monitoring system based on the ACCT [37] (Bergoz Instrumentation, Saint‐Genis‐Pouilly, France).

### 5.13| FLASH‐RT in CAM Models

The parameters listed in Table S2 were applied to all in vivo irradiations. The linear accelerator was operated in a horizontal configuration, with the eggs positioned on a PMMA holder to maintain their alignment in front of the applicator. Due to the eggs’ dimensions, achieving adequate dose coverage of the target area (where the embryo is located) with a single field was not feasible, so two opposing fields were employed. Dose distributions for both single‐field and dual‐field configurations were estimated using Monte Carlo simulations (Figure S8). Although using two fields introduced a 1‐min delay between exposures—meaning the total irradiation time did not satisfy FLASH criteria (<0.2 s)—each individual field still complied with the previously established parameters.

### 5.13 CAM Assay

CAM models with SCC‐25 cells have been produced through the optimized protocol [24]. Fertilized red Leghorn eggs were purchased from a local supplier and immediately stored at 4°C upon delivery. Before incubation, at EDD0, the eggs were cleaned with deionized water and placed in trays inserted in a fan‐assisted incubator (FIEM MG 140/200) set at 37.5°C with ≈47% humidity. Eggs were punctured on EDD3 and on EDD6 a small window of 1 cm^2^ was made to distinguish fertilized and not fertilized eggs and properly inoculate tumor cells on the CAM. For the grafting procedure, 2 × 10^6^ cells/egg were diluted in a 1:1 mixture of Matrigel (Corning; Ref 35423) and serum‐free cell culture medium for a final volume of 25 µL per egg. Eggs were incubated for 4 days, and tumor‐bearing embryos were randomized at EDD10 and divided into four groups of treatment. FLASH‐RT (4 Gy) was delivered at EDD10 and ARN‐24139 (10 µM) at EDD12. Tumors were photographed until EDD15 using a portable digital microscope (DinoLite) and their area was determined by drawing a precise ROI around the tumor with ImageJ software. On EDD15 the experiments were concluded, tumors harvested and fixed with formalin. All CAM experiments were conducted in accordance with institutional guidelines and applicable national regulations for the use of chick embryos in research. The experimental procedures were terminated before hatching and performed within the developmental time window commonly considered outside the scope of animal experimentation regulations.

### 5.14 Immunohistochemistry

Deparaffinized samples were treated for 10 min with a hydrogen peroxide solution (3%) to stop endogenous peroxidase activity. Thereafter, samples were immersed in ethylene diamine triacetic acid‐based buffer (pH 8.0; Leica Biosystems RE7116‐CE) and placed in a microwave oven (10 min, 480 W) for antigen retrieval. Nonspecific staining was prevented with blocking peptide (Abcam HRP/DAB; detection kit ab 64261). The primary antibodies for Ki67 (rabbit monoclonal Invitrogen MA5–14520, diluted 1:100), CC‐3 (rabbit polyclonal Cell Signaling Technology; 9661S; diluted 1:500) and Phospho‐S139 Histone H2A.X ([EP854(2)Y] (rabbit monoclonal ab81299, diluted 1:2000) were applied and left overnight at 4°C. The detection was performed using the streptavidin–biotin technique (Abcam HRP/DAB; detection kit, ab 64261). Finally, the chromogen (diaminobenzidine) was used for IHC development and Mayer's hematoxylin for counterstaining. To quantitatively determine the protein expression, IHC images were processed with Aperio ImageScope software, which detected the signal intensity. The positivity was determined by averaging at least five different areas of the slide section and data are reported as fold change over control.

### 5.15 Statistical Analysis

All quantitative data are presented as mean ± SD unless otherwise indicated. Unless otherwise specified, all experiments were performed using three independent biological replicates, each analyzed with the technical replicates described in the corresponding Methods or figure legends. Statistical analyses were performed using GraphPad Prism version 9.0 (GraphPad Software, San Diego, CA, USA). Comparisons between experimental groups were performed using one‐way or two‐way analysis of variance (ANOVA) followed by Tukey's multiple comparisons test, as appropriate for each experiment. The specific statistical test applied to each dataset is indicated in the corresponding figure legends. Statistical significance was defined as *p* ≤ 0.05.

## Author Contributions

P.S., N.G., G.G., and A.G.: in vitro 2D experiments. P.S., M.S., and V.F.: in vitro 3D experiments. P.S., F.P., V.F., N.G., and G.G.: in vivo experiments. A.C., M.C., and F.D.M.: irradiation procedures. M.M.: immunohistochemistry analysis, A.M., and A.G.L.D.R.: FACS analysis. N.B. and M.N.: chemical synthesis. P.S., M.D.V., F.P., and V.V.: data interpretation, project design and coordination. P.S.: manuscript writing. All authors have discussed the data. All authors have read, revised, and approved the final manuscript.

## Funding

P.S. thanks Associazione Italiana per la Ricerca sul Cancro (AIRC), Project Code: 29543. M.D.V. thanks AIRC for financial support (IG 30631).

## Ethics Statement

Experiments involving chick CAM models were conducted in accordance with applicable institutional and national guidelines. Since the experiments were completed before hatching, no specific ethical approval for animal experimentation was required according to current regulations.

## Conflicts of Interest

M.D.V. is coinventor of patent application WO2018167187A1/PCT/EP2018/056469, entitled “5‐carboxamide‐2‐thiobarbituric acids and use thereof as medicaments,” filed by Università degli Studi di Padova and Fondazione Istituto Italiano di Tecnologia; the listed inventors are Marco De Vivo, José Antonio Ortega Martinez, Claudia Sissi, and José Manuel Arencibia Jimenez. The WO application is listed as ceased, with related EP and US family members reported as active/granted; the patent covers the class of Topo‐II inhibitor compounds related to ARN‐24139 investigated in this manuscript. All the other authors declare no competing financial interests that could have appeared to influence the work reported in this paper.

## Supporting information




**Supporting File 1**: mco270937‐Supp‐0001‐SuppMatt.docx

## Data Availability

All data generated and analyzed during this study are included in this published article and its supplementary information files. The raw data are available from the corresponding author on reasonable request.
