# Supplementary material for: Antitumor Activity of Combined Topoisomerase Inhibition and FLASH Radiotherapy in Head and Neck Carcinoma Biomodels
Source: MedComm (2020). 2026 Sep 2;7(9):e70937. doi: 10.1002/mco2.70937 (PMC13535334; doi:10.1002/mco2.70937)
Supplement: Supplementary file 1 [file MCO2-7-e70937-s001.docx]

**Supplementary Information**

**Antitumor Activity of Combined Topoisomerase Inhibition and FLASH Radiotherapy in Head and Neck Carcinoma Biomodels**

Patrizia Sarogni^1,2,^*****, Nicoletta Brindani^1^, Valentina Frusca^2,3^, Melissa Santi^4^, Andrea Cavalieri^5,6^, Mariagrazia Celentano^6,7^, Michele Menicagli^8^, Andrea Marranci^8^, Andrea Ghelli Luserna di Rorà^8^, Federico Purificato^2,9^, Michela Nigro^1^, Noemi Giannini^2,10^, Alessandra Gonelli^2,10^, Giovanni Gadducci^2,10^, Fabio Di Martino^6,11^, Fabiola Paiar^#,10^, Marco De Vivo^1,#,^*****, Valerio Voliani^2,12,13,#,^*****

^1^ Molecular Modeling & Drug Discovery Lab, Istituto Italiano di Tecnologia, Via Morego, 30 – Genoa, 16163, Italy

^2^ Center for Nanotechnology Innovation@NEST – Istituto Italiano di Tecnologia, Piazza San Silvestro 12, 56127, Pisa, Italy

^3^ Scuola Superiore Sant’Anna, Piazza Martiri della Libertà 33, 56127 Pisa, Italy

^4^ Istituto Nanoscienze-CNR and NEST Laboratory, Piazza San Silvestro 12, 56127 Pisa, Italy

^5^ Center for Instrument Sharing of the University of Pisa (CISUP), University of Pisa, Italy

^6^ Centro Pisano ricerca e implementazione clinica FLASH Radiotherapy (CPFR@CISUP), Presidio S. Chiara, via Roma

^7^ National Institute of Nuclear Physics (INFN), Largo B. Pontecorvo, 3, Pisa, 56127, Italy

^8^ Fondazione Pisana per la Scienza ONLUS, via Ferruccio Giovannini, 13 - San Giuliano Terme (56017), Italy

^9^ NEST – Scuola Normale Superiore, Piazza San Silvestro, 12 - Pisa (56126), Italy

^10^ Radiation Oncology Unit, Pisa University Hospital, Via Roma 67, 56126, Pisa, Italy

^11^ Unit of Medical Physics, Pisa University Hospital “Azienda Ospedaliero-Universitaria

Pisana”, Pisa, Italy

^12^ Department of Pharmacy, University of Genoa, Viale Cembrano, 4 – Genoa, 16148, Italy

^13^ Centro 3R – Centro Interuniversitario per la promozione dei principi delle 3R nella didattica e nella ricerca.

***** Corresponding authors: [patrizia.sarogni@iit.it](mailto:patrizia.sarogni@iit.it); [valerio.voliani@unige.it](mailto:valerio.voliani@unige.it); [marco.devivo@iit.it](mailto:marco.devivo@iit.it)

^#^ Shared senior authorship

**Supplementary Figure 1**

**Supplementary Figure 1:** Screening of others custom-designed topoisomerase II inhibitors drugs. **A)** SCC-25 cells were treated at increased concentration with the respective compounds for 24h and cell viability was measured up to 72 h. Data were normalized over the viability of control treated with medium. Data is reported as mean ± SD of three experimental replicates. B) Etoposide and Merbarone were tested at single concentrations (25 μM). Viability was measured up to 72 h and data normalized over the control treated with medium set as 100% (dashed line). Data is reported as mean ± SD of three biological experiments, each with three experimental replicates.

**Supplementary Figure 2**

**Supplementary Figure 2:** Comparative cytotoxicity of cisplatin and ARN-24139 in SCC-25 and healthy HBEpC cells. SCC-25 and HBEpC cells were treated with increasing concentrations of cisplatin under the same experimental conditions used for ARN-24139 treatment. Cell viability was assessed using the WST-8 assay and normalized to untreated controls. Data are presented as mean ± SD of three independent experiments performed in triplicate. Statistical analyses were performed using two-way ANOVA followed by Tukey’s multiple comparison test.

**Supplementary Figure 3**

**Supplementary Figure 3:** Evaluation of different combination treatment modality. A) SCC-25 cells were treated with ARN-24139 (5 μM) for 24 h and then irradiated with increased FLASH-RT dose. Viability was monitored up to 8 days. Data is reported as mean ± SD of three experimental replicates. B) SCC-25 cells were concomitantly treated with ARN-24139 and FLASH-RT and viability monitored up to 8 days. Data is reported as mean ± SD of three experimental replicates.

**Supplementary Figure 4**

**Supplementary Figure 4:** Dual treatment in three-dimensional tumor spheroids. SCC-25 3D models were treated with FLASH-RT at increased doses and ARN-24139 (12 μM) was administered after 48 h. No effect was observed with respect to 2D models. Data is reported as mean ± SD of three experimental replicates.

**Supplementary Figure 5**

**Supplementary Figure 5:** Antitumor efficacy of CONV-RT combined with ARN-24139 in 2D SCC-25 cells. A) SCC-25 cells were exposed to increasing doses of CONV-RT and treated with ARN-24139 (5 μM) 48 h after irradiation. Cell viability was monitored up to 8 days post-irradiation. A significant reduction in cell viability was observed for the combined treatment compared with CONV-RT alone at the lowest radiation dose (4 Gy) after 8 days. Data are presented as mean ± SD of three independent biological experiments, each performed in triplicate. Statistical significance was determined using two-way ANOVA followed by Tukey's multiple comparison test (***p < 0.001). (B) Drug–radiation interactions were evaluated using the SynergyFinder online platform (https://synergyfinder.org). Red areas indicate synergistic interactions, whereas green areas indicate antagonistic interactions. The highest synergy score was observed at 8 days post-treatment, consistent with the viability results.

**Supplementary Figure 6**

**Supplementary Figure 6:** Gene expression profile. A) Tumor (red) and normal (black) differential expression analysis of TOP2A gene in HNSCC patients. Data is reported as Log_2_ Transcript Per Milion +1. B) Patient survival analysis according to low and high expression of TOP2A gene. GEPIA, a web server for cancer and normal gene expression profiling and interactive analyses, was used.

**Supplementary Figure 7**

**Supplementary Figure 7:** *Left:* Vertical setup of the Electron Flash. *Right:* Example of a Petri dish positioned on the solid water slab. On the Petri side is positioned the flashDiamond detector to correctly monitor the dose output.

**Supplementary Figure 8**

**Supplementary Figure 8:** DVH for the embryo for single field (dashed line) and two opposing fields (solid line) configurations. As shown, the dose deposition using two opposing electron fields results in better target coverage than the single field deposition.

**Supplementary Table S1:** Human topoIIα inhibitory activities measured by decatenation assay and antiproliferative activity results in three human cancer cell lines. Values show the IC50 ± standard deviation. Each experiment has been repeated twice.

| **Compound** | **topoIIα IC_50_ (*µ*M)** | **HELA IC_50_ (*µ*M)** | **DU145 IC_50_ (*µ*M)** | **A549 IC_50_ (*µ*M)** |
| --- | --- | --- | --- | --- |
| Etoposide | 31.6 ± 0.2 | 13.3 ± 1 | 0.6 ± 0.06 | 1.3 ± 0.1 |
| ARN24139^a^ | 7.3 ± 1.5 | 3.8 ± 0.3 | 4.7 ± 0.1 | 3.1 ± 0.1 |
| ARN-25656 | 9.5 ± 2.8 | 8.1 ± 3.4 | 15.5 ± 3.4 | 39.2 ± 1.3 |
| ARN-26009 | 1.5 ± 0.7 | 7.7 ± 2.4 | 3.1 ± 1.4 | 7.6 ± 3.5 |
| ARN-26010 | 18.2 ± 8.9 | 8.7 ± 0.8 | 4.5 ± 3.5 | 14.2 ± 1.3 |
| ARN-26672 | 4.2 ± 0.2 | 20.5 ± 3.8 | 4.6 ± 2.3 | 15.6 ± 1.7 |
| ARN-26673 | 7.5 ± 3.7 | 16.4 ± 12.0 | 14.5^b^ | 16.1 ± 4.7 |

a) The data has been taken from Arencibia et al.^1^
b) IC_50_ value obtained from a single measurement

**Supplementary Table S2:** Physical parameters for FLASH and CONV irradiation

| Irradiation modality | Dose per pulse [Gy] | Average dose rate | Average dose within a pulse | Pulse duration [µs] |
| --- | --- | --- | --- | --- |
| FLASH | 1 | 240 Gy/s | 2.5 x 10^5^ Gy/s | 4 µs |
| CONV | 0.02 | 6 Gy/min | 5 x 10^3^ Gy/s | 4 µs |

**Supplementary information – CHEMISTRY**

**General Considerations**

All the commercially available reagents and solvents were used as purchased from vendors without further purification. Dry solvents were purchased from Sigma-Aldrich. Automated column chromatography purifications were done using a Teledyne ISCO apparatus (CombiFlash® Rf) with pre-packed silica gel columns of different sizes (from 4 g up to 24 g) and mixtures of increasing polarity of cyclohexane and ethyl acetate (EtOAc) or dichloromethane (DCM) and methanol (MeOH). NMR data were collected on 400 MHz or 600 MHz (1H) and 100 MHz or 150 MHz (^13^C). Spectra were acquired at 300 K, using deuterated dimethylsulfoxide (DMSO–*d6*) or deuterated chloroform (CDCl_3_) as solvents. For ^1^H-NMR, data are reported as follows: chemical shift, multiplicity (s= singlet, d= doublet, dd= double of doublets, t= triplet, q= quartet, m= multiplet), coupling constants (Hz) and integration. UPLC/MS analyses were run on a Waters ACQUITY UPLC/MS instrument consisting of a SQD (Single Quadrupole Detector). The analyses were performed on an ACQUITY UPLC BEH C_18_ column (50x2.1mmID, particle size 1.7µm) with a VanGuard BEH C_18_ pre-column (5x2.1mmID, particle size 1.7µm) (LogD>1). The mobile phase was 10mM NH_4_OAc in H_2_O at pH 5 adjusted with AcOH (A) and 10mM NH_4_OAc in MeCN-H_2_O (95:5) at pH 5 (B). Electrospray ionization in positive and negative mode was applied in the mass scan range 100-500Da. Depending on the analysis method used, a different gradient increasing the proportion of mobile phase B was applied. For analysis method 1, the mobile-phase B proportion increased from 5 % to 95 % in 3 min. For analysis method 2, the mobile-phase B proportion increased from 50 % to 100 % in 3 min. The analysis of QC was performed starting from a10mM stock solution of test compound in DMSO-d6 and further diluted 20-fold with CH_3_CN-H2O (1:1) for analysis. The QC analyses were performed on a Waters ACQUITY UPLC/MS system consisting of a SQD (Single Quadrupole Detector) Mass Spectrometer equipped with an Electrospray Ionization interface and a Photodiode Array Detector. Electrospray ionization in positive and negative mode was applied in the mass scan range 100-500Da. The PDA range was 210-400nm. The analyses were run on an ACQUITY UPLC BEH C18 column (100x2.1mmID, particle size 1.7µm) with a VanGuard BEH C18 pre-column (5x2.1mmID, particle size 1.7µm). The mobile phase was 10mM NH4OAc in H2O at pH 5 adjusted with AcOH (A) and 10mM NH4OAc in CH3CN-H2O (95:5) at pH 5 (B) with 0.5mL/min as flow rate. A linear gradient was applied: 0-0.2min: 10%B, 0.2-6.2min: 10-90%B, 6.2-6.3min: 90-100%, 6.3-7.0min: 100%B (QC method). All final compounds displayed ≥95% purity as determined by UPLC/MS analysis (unless otherwise indicated). Compound **1** was synthesized as previously reported by Ortega et al.^11^

**Scheme S1: Synthesis of Topo-II inhibitors**

*Reaction conditions:* a) anilines R-NH_2_ **2a-e**, toluene dry, 100°C; b) HCl (4M in 1,4-dioxane dry), 1,4-dioxane dry.

**Scheme S2: Synthesis of aniline of 2b-e.**

*Reaction conditions:* a) proper alkyl bromide, K_2_CO_3_, TBAI, CH_3_CN dry, 80°C; b) Et_3_SiH, Pd/C, EtOH, rt; c) Boc_2_O, DMAP, DIPEA, DCM dry; d) MeI, K_2_CO_3_, DMF dry.

**General procedure 1: amidation of ester 1 with aniline of type 2**. A solution 0.1 M of ethyl 6-hydroxy-4-oxo-1,3-diphenyl-2-thioxo-1,2,3,4-tetrahydropyrimidine-5-carboxylate **1** (1 equiv) and an appropriate amine (1.1 equiv) in toluene dry was stirred at 100 °C for 2 h. After completion of reaction, the solvent was removed under vacuum. The product was purified by flash chromatography and/or by trituration with MeOH, yielding pure compound.

**General procedure 2: Boc removal**. A solution 0.05 M of compounds **3d-e**, **8a-b** (1 equiv) in 1,4-dioxane dry was treated with HCl (4M in 1,4 dioxane dry) (5 equiv) and stirred at room temperature for 20h. After completion of reaction, the solvent was removed under vacuum. The product was purified by trituration with Et_2_O, yielding pure compound.

**General procedure 3: reduction of nitroarene to aniline**. Et_3_SiH (5 equiv) was slowly added to a suspension of compound of type **5** and **8** (1 equiv) and Pd/C (8% w/w) in EtOH (0.3M) under argon, and reaction mixture stirred at room temperature for 6 hours. After that, the mixture was filtered through a pad of celite, rised with EtOH, and concentrated under vacuum. Purification by normal phase chromatography afforded pure desired compounds.

**General procedure 4: alkylation.** To a well-stirred solution of suitable phenol **4** (1 equiv) and K_2_CO_3_ (2 equiv) in CH_3_CN dry (0.5 M) was added suitable alkyl bromide (2 equiv) and TBAI (0.5 equiv). and the reaction mixture heated at 85°C and stirred for 5 hours under argon**.** After that NH_4_Cl sat aq sol was added, and aqueous layer was extracted with EtOAc. Collected organic layers were dried over Na_2_SO_4_, filtered, and concentrated under vacuum. Final purification by normal phase chromatography afforded pure desired compounds of type **16e-l**.

**General procedure 5: Boc protection.** DMAP (0.2 equiv) and DIPEA (1.5 equiv) were added to a 0.2 M solution of compound of type **6** (1 equiv) in DCM dry under argon. Reaction mixture stirred for 30 minutes at room temperature, after that Boc2O (1.2 equiv) was added and reaction mixture stirred for 6 hours. So NH_4_Cl sat aqueous solution was added until pH = 7, and aqueous layer was extracted with DCM. Collected organic layers were dried over Na_2_SO_4_, filtered, and concentrated under vacuum. Purification by normal phase chromatography afforded desired compounds.

**Synthesis of final Topo-II inhibitors (Scheme S1)**

**6-hydroxy-4-oxo-1,3-diphenyl-2-thioxo-N-(3,4,5-trichlorophenyl)- 1,2,3,4 –tetrahydro pyrimidine-5-carboxamide (3a, ARN-25656, Scheme S1)**. The title compound was prepared according to general procedure 1 using intermediate aniline **2a** (41.1 mg, 0.21 mmol), ester **1** (70.0 mg, 0.19 mmol) in toluene dry (1.90 mL). Trituration of the residue with MeOH (1.9 mL) afforded pure compound 13 (14.6 mg, 15% yield). UPLC/MS: Rt = 2.31 min (method 1). MS (ESI) m/z: 518.0/519.9/521.9 [M + H]^+^, C_23_H_15_Cl_3_N_3_O_3_S [M + H]^+^ calculated, 519.9. HRMS (ESI) m/z: 517.9894 calcd for C_23_H_14_Cl_3_N_3_O_3_S [M+H]+; found m/z: 517.9898. 1H NMR showed the presence of two isomers (tautomers or conformers) Ia/Ib in a 74/25 ratio. ^1^H NMR (101 MHz, DMSO-d6) of major isomer: δ 12.14 (s, 1H), 7.86 (s, 2H), 7.42 – 7.38 (m, 4H), 7.32 – 7.28 (m, 2H), 7.23 – 7.17 (m, 4H). ^13^C NMR (101 MHz, DMSO-d_6_) of major isomer: δ 178.4 (Cq), 166.1 (Cq), 163.2 (Cq), 140.8 (Cq, 2C), 139.0 (Cq), 132.6 (Cq, 2C), 129.3 (CH, 4C), 128.7 (CH, 4C), 127.5 (CH, 2C), 122.5 (Cq), 120.0 (CH, 2C), 88.5 (Cq).

**6-hydroxy-4-oxo-1,3-diphenyl-N-(4-propoxyphenyl)-2-thioxo-1,2,3,4-tetrahydropyrimidine-5-carboxamide (3b, ARN-26009, Scheme S1).** The title compound was prepared according to general procedure 1 using aniline **2b** (16.0 mg, 0.11 mmol), ester **1** (37 mg, 0.10 mmol) in toluene dry (1.00 mL). Trituration of the residue with MeOH (1 mL) afforded pure compound 1 (22.0 mg, 47% yield). UPLC/MS: Rt = 2.22 min (method 1). MS (ESI) m/z:.474.0 [M + H]^+^, C_26_H_2_N_3_O_4_S [M + H]^+^ calculated, 474.5. HRMS (ESI) m/z: 474.1482 calcd for C_26_H_23_N_3_O_4_S [M+H]^+^; found m/z: 474.1492. ^1^H NMR (101 MHz, DMSO-d6): δ 11.54 (s, 1H), 7.53 – 7.37 (m, 8H), 7.37 – 7.26 (m, 4H), 7.00 – 6.90 (m, 2H), 3.91 (t, J = 6.5 Hz, 2H), 1.71 (sext, J = 7.1 Hz, 2H), 0.96 (t, J = 7.4 Hz, 3H). ^13^C NMR (101 MHz, DMSO-d_6_) δ 178.4 (Cq), 168.3 (Cq), 164.3 (Cq), 156.8 (Cq), 139.2 (Cq, 2C), 129.2 (CH, 4C), 129.0 (CH, 4C), 128.4 (CH, 2C), 123.9 (CH, 2C), 115.0 (CH, 2C), 83.8 (Cq), 69.3 (CH2), 22.0 (CH2), 10.4 (CH3).

**N-(4-(sec-butoxy) phenyl)-6-hydroxy-4-oxo-1,3-diphenyl-2-thioxo- 1,2,3,4- tetrahydro pyrimidine -5- carboxamide (3c, ARN-26010, Scheme S1).** The title compound was prepared according to general procedure 1 using aniline **2c** (18.2 mg, 0.11 mmol), ester **1** (37 mg, 0.10 mmol) in toluene dry (1.00 mL). Trituration of the residue with MeOH (1 mL) afforded pure compound ARN-25010 (25.3 mg, 51% yield). UPLC/MS: Rt = 2.29 min (method 1). MS (ESI) m/z:.488.1 [M + H]^+^, C_27_H_26_N_3_O_4_S [M + H]^+^ calculated, 488.6. HRMS (ESI) m/z: 488.1639 calcd for C_27_H_25_N_3_O_4_S [M+H]^+^; found m/z: 488.1638. ^1^H NMR (101 MHz, DMSO-*d_6_*): δ 11.5 (s, 1H), 7.5 – 7.46 (m, 4H), 7.45 – 7.36 (m, 4H), 7.34 – 7.32 (4H), 6.98 – 6.88 (m, 2H), 4.35 (sext, J = 6.1 Hz, 1H), 1.69 – 1.50 (m, 2H), 1.20 (d, J = 6.0 Hz, 3H), 0.90 (t, J = 7.4 Hz, 3H). ^13^C NMR (101 MHz, DMSO-*d_6_*) δ 178.4 (Cq), 168.3 (Cq), 156.0 (Cq), 165.2 (Cq, broad signal), 139.2 (Cq, 2C), 129.2 (CH, 4C), 129.0 (CH, 2C), 128.4 (CH, 2C), 124.0 (CH, 2C), 116.1 (CH, 2C), 83.8 (Cq), 74.6 (CH), 28.6 (CH_2_), 19.1 (CH_3_), 9.6 (CH_3_).

**tert-butyl (3-fluoro-5-(6-hydroxy-4-oxo-1,3-diphenyl-2-thioxo- 1,2,3,4 –tetrahydro pyrimidine-5-carboxamido) phenyl) (methyl)carbamate (3d, Scheme S1)**. The title compound was prepared according to general procedure 1 using intermediate aniline **2d** (54.0 mg, 0.22 mmol), ester **1** (75.0 mg, 0.20 mmol) in toluene dry (2.00 mL). Trituration of the residue with MeOH (2.0 mL) afforded pure compound **3d** (70.5 mg, 57% yield). UPLC/MS: Rt = 2.15 min (method 1). MS (ESI) m/z: 563.1 [M + H]^+^, C_29_H_28_FN_4_O_5_S [M + H]^+^ calculated, 563.6. ^1^H NMR (101 MHz, DMSO-*d_6_*): δ 11.68 (s, 1H), 7.50 – 7.45 (m, 4H), 7.42 – 7.34 (m, 3H), 7.32 – 7.29 (m, 5H), 7.01 (dt, J = 10.6, 2.3 Hz, 1H), 3.17 (s, 3H), 1.39 (s, 9H).

**N-(3-fluoro-5-(methylamino) phenyl)-6-hydroxy-4-oxo-1,3-diphenyl-2-thioxo- 1,2,3,4 –tetrahydro pyrimidine-5-carboxamide (ARN-26672, Scheme S1)**. The title compound was prepared according to general procedure 2 using intermediate **3d** (56.3 mg, 0.10 mmol) and HCl (4 M in 1,4-dioxane) (0.13 mL) in 1,4-dioxane dry (2.0 mL). Trituration of the residue with Et_2_O (1.0 mL) afforded pure compound ARN-26672 (29.1 mg, 63% yield). UPLC/MS: Rt = 1.93 min (method 1). MS (ESI) m/z:.463.0 [M + H]^+^, C_24_H_20_FN_4_O_3_S [M + H]^+^ calculated, 463.5. HRMS (ESI) m/z: 463.1235 calcd for C_24_H_19_FN_4_O_3_S [M+H]^+^; found m/z: 463.1239. ^1^H NMR (101 MHz, DMSO-*d_6_*): δ 11.60 (s, 1H), 7.51 – 7.47 (m, 4H), 7.44 – 7.36 (m, 2H), 7.36 – 7.18 (m, 4H), 6.67 (dt, J = 10.5, 1.9 Hz, 1H), 6.51 (t, J = 2.0 Hz, 1H), 6.23 (dt, J = 11.4, 2.1 Hz, 1H), 2.66 (s, 3H). 13C NMR (101 MHz, DMSO-*d_6_*) δ 178.7 (Cq), 169.2 (Cq, 2C), 163.8 (Cq, d, ^1^J_CF_ = 238.2 Hz), 152.1 (Cq, d, ^3^J_CF_ = 13.3 Hz), 139.4 (Cq, 2C), 137.5 (Cq, d, ^3^J_CF_ = 15.1 Hz), 129.6 (CH, 4C), 129.3 (CH, 4C), 128.9 (CH, 2C), 101.4 (CH), 96.6 (CH), 96.5 (CH, d, ^2^J_CF_ = 27.5 Hz), 96.3 (CH, d, ^2^J_CF_ = 24.5 Hz), 84.5 (Cq), 40.7 (CH_3_). ^19^F NMR (376 MHz, DMSO-*d_6_*) δ −111.9 (s).

**tert-butyl (3-chloro-5-(6-hydroxy-4-oxo-1,3-diphenyl-2-thioxo- 1,2,3,4 –tetrahydro pyrimidine-5-carboxamido) phenyl)(methyl)carbamate (3e, Scheme S1)**. The title compound was prepared according to general procedure 1 using intermediate aniline **2e** (57.2 mg, 0.22 mmol), ester **1** (75.0 mg, 0.20 mmol) in toluene dry (2.00 mL). Trituration of the residue with MeOH (2.0 mL) afforded pure compound **3e** (60 mg, 51% yield). UPLC/MS: Rt = 2.25 min (method 1). MS (ESI) m/z: 577.2/579.2 [M − H]^−^, C_29_H_26_ClN_4_O_5_S [M + H]^+^ calculated, 578.1. ^1^H NMR (101 MHz, DMSO-*d_6_*): δ 11.65 (s, 1H), 7.55 (t, J = 1.9 Hz, 1H), 7.50 – 7.46 (m, 4H), 7.44 (t, J = 2.0 Hz, 1H), 7.42 – 7.36 (m, 2H), 7.35 – 7.28 (m, 4H), 7.22 (t, J = 1.9 Hz, 1H), 3.17 (s, 3H), 1.39 (s, 9H).

**N-(3-chloro-5-(methylamino) phenyl)-6-hydroxy-4-oxo-1,3-diphenyl-2-thioxo- 1,2,3,4 –tetrahydropyrimidine-5-carboxamide (ARN-26673, Scheme S1)**. The title compound was prepared according to general procedure 2 using intermediate **3e** (46.4 mg, 0.08 mmol) and HCl (4 M in 1,4-dioxane) (0.10 mL) in 1,4-dioxane dry (1.6 mL). Trituration of the residue with Et_2_O (1.0 mL) afforded pure compound ARN-26673 (19.6 mg, 51% yield). UPLC/MS: Rt = 1.99 min (method 1). MS (ESI) m/z:.479.0 [M + H]^+^, C_24_H_20_ClN_4_O_3_S [M + H]^+^ calculated, 479.9. HRMS (ESI) m/z: 479.0939 calcd for C_24_H_19_ClN_4_O_3_S [M+H]^+^; found m/z: 479.094. ^1^H NMR (101 MHz, DMSO-*d_6_*): δ 11.57 (s, 1H), 7.52 – 7.47 (m, 4H), 7.44 – 7.37 (m, 2H), 7.36 – 7.28 (m, 4H), 6.86 (t, J = 1.9 Hz, 1H), 6.60 (t, J = 2.0 Hz, 1H), 6.45 (t, J = 2.0 Hz, 1H), 2.65 (s, 3H). ^13^C NMR (101 MHz, DMSO-*d_6_*) δ 178.2 (Cq), 168.8 (Cq), 164.4 (Cq), 151.2 (Cq), 138.9 (Cq, 2C), 137.1 (Cq), 134.0 (Cq), 129.2 (CH, 4C), 128.8 (CH, 4C), 128.4 (CH, 2C), 109.0 (CH), 108.6 (CH), 103.4 (CH), 84.0 (Cq), 66.3 (CH_3_).

**Synthesis of aniline starting materials (Scheme S2).**

**1-nitro-4-propoxybenzene (5a, Scheme S2 – equation a)**. The title compound was prepared according to general procedure 4 using phenol **4** (300.0 mg, 2.15 mmol), K_2_CO_3_ (596.0 mg, 4.31 mmol), 1-bromopropane (0.39 mL, 4.31 mmol), TBAI (398.0 mg, 1.08 mmol) in CH_3_CN dry (4.3 mL). Final normal phase purification (elution by gradient from 100% to 85/15 cyclohexane/EtOAc) afforded pure title compound **5a** (383.0 mg, 98% yield). UPLC/MS: Rt = 2.34 min (method 1), no ionization. ^1^H NMR (400 MHz, CDCl_3_) δ 8.20 – 8.18 (m, 2H), 6.95 – 6.93 (m, 2H), 4.01 (t, J = 6.5 Hz, 2H), 1.85 (sextet, J = 7.1 Hz, 2H), 1.06 (t, J = 7.4 Hz, 3H).

**4-propoxyaniline (2b, Scheme S2 – equation a).** The title compound was prepared according to general procedure 3 using intermediate aniline **5a** (385.0 mg, 2.12 mmol), Et_3_SiH (1.69 mL, 10.60 mmol), Pd/C (30.8 mg) in EtOH (7.1 mL). Final normal phase purification (elution by gradient from 100% to 45/55 cyclohexane/EtOAc) afforded pure title compound **2b** (288.0 mg, 90% yield). UPLC/MS: Rt = 1.69 min (method 1). MS (ESI) m/z: 151.9 [M + H]^+^, C_9_H_14_NO [M + H]^+^ calculated, 151.2. ^1^H NMR (101 MHz, DMSO-*d_6_*) δ 6.67 – 6.58 (m, 2H), 6.52 – 6.45 (m, 2H), 4.55 (s, 2H), 3.75 (t, J = 6.5 Hz, 2H), 1.64 (sext, J = 7.1 Hz, 2H), 0.93 (t, J = 7.4 Hz, 3H).

**1-(sec-butoxy)-4-nitrobenzene (5b, Scheme S2 – equation a)**. The title compound was prepared according to general procedure 4 using phenol **4** (300.0 mg, 2.15 mmol), K_2_CO_3_ (596.0 mg, 4.31 mmol), 2-bromobutane (0.47 mL, 4.31 mmol), TBAI (398.0 mg, 1.08 mmol) in CH_3_CN dry (4.3 mL). Final normal phase purification (elution by gradient from 100% to 90/10 cyclohexane/EtOAc) afforded pure title compound **20c** (299.0 mg, 71% yield). UPLC/MS: Rt = 2.46 min (method 1), no ionization. δ 1H NMR (400 MHz, CDCl_3_) δ 8.23 – 8.13 (m, 2H), 6.96 – 6.87 (m, 2H), 4.42 (sextet, J = 6.1 Hz, 1H), 1.84 – 1.73 (m, 1H), 1.71 – (m, 1H), 1.34 (d, J = 6.1 Hz, 3H), 0.99 (t, J = 7.4 Hz, 3H).

**4-(sec-butoxy) aniline (2c, Scheme S2 – equation a)**. The title compound was prepared according to general procedure 3 using intermediate **5b** (300.0 mg, 1.54 mmol), Et_3_SiH (1.23 mL, 7.70 mmol), Pd/C (24.0 mg) in EtOH (5.1 mL). Final normal phase purification (elution by gradient from 100% to 60/40 cyclohexane/EtOAc) afforded pure title compound **2c** (223.6 mg, 88% yield). UPLC/MS: Rt = 1.84 min (method 1). MS (ESI) m/z: 165.9 [M + H]^+^, C_10_H_16_NO [M + H]^+^ calculated, 166.2. ^1^H NMR (101 MHz, DMSO-*d_6_*) δ 6.65 – 6.58 (m, 2H), 6.51 – 6.44 (m, 2H), 4.57 (s, 2H), 4.06 (sext, J = 6.0 Hz, 1H), 1.64 – 1.40 (m, 2H), 1.13 (d, J = 6.1 Hz, 3H), 0.89 (t, J = 7.4 Hz, 3H).

**tert-butyl (3-fluoro-5-nitrophenyl) carbamate (7a, Scheme S2 – equation b).** The title compound was prepared according to general procedure 5 using nitroaniline **6a** (312.0 mg, 2.0 mmol), DIPEA (0.53 mL, 3.00 mmol), DMAP (50.0 mg, 0.40 mmol), and Boc_2_O (524 mg, 2.40 mmol) in DCM (10 mL). Final normal phase purification afforded pure compound **7a** (230.6 mg, 45% yield). UPLC/MS: Rt = 1.33 min (method 2), MS (ESI) m/z: 255.0 [M − H]^−^, C_11_H_12_FN_2_O_4_ [M − H]^−^ calculated, 255.2. ^1^H NMR (101 MHz, DMSO-*d_6_*): δ 10.11 (s, 1H), 8.25 (t, J = 2.2 Hz, 1H), 7.73 – 7.68 (m, 1H), 7.67 (d, J = 2.0 Hz, 1H), 1.49 (s, 9H).

**tert-butyl (3-fluoro-5-nitrophenyl) (methyl)carbamate (8a, Scheme S2 – equation b).** The title compound was prepared according to general procedure 4 using compound **7a** (230.0 mg, 0.90 mmol), K_2_CO_3_ (248.4 mg, 1.80 mmol), iodoethane (0.13 mL, 1.80 mmol), in DMF dry (0.8 mL). Final normal phase purification (elution by gradient from 100% to 90/10 cyclohexane/EtOAc) afforded pure title compound **8a** (175.0 mg, 72% yield). UPLC/MS: Rt = 1.42 min (method 2), MS (ESI) m/z: 214.9 [M – ^t^Bu + H]^+^, C_8_H_8_FN_2_O_4_ [M + H]^+^ calculated, 214.2. ^1^H NMR (400 MHz, DMSO-*d_6_*) δ 8.10 (t, J = 2.3 Hz, 1H), 7.88 (dt, J = 8.5, 2.2 Hz, 1H), 7.74 (dt, J = 10.5, 2.2 Hz, 1H), 3.27 (s, 3H), 1.44 (s, 9H).

**tert-butyl (3-amino-5-fluorophenyl) (methyl)carbamate (2d, Scheme S2 – equation b)**. The title compound was prepared according to general procedure 3 using intermediate **8a** (270 mg, 1.00 mmol), Pd/C (13.6 mg), Et_3_SiH (0.80 mL, 5.00 mmol), in EtOH (3.3 mL). Final normal phase purification (elution by gradient from 100% to 80/20 cyclohexane/EtOAc) afforded pure title compound **2d** (182.6 mg, 76% yield). UPLC/MS: Rt = 1.88 min (method 1). MS (ESI) m/z: 184.9 [M − *^t^*Bu + H]^+^, C_11_H_17_FN_2_O_2_ [M − *^t^*Bu + H]^+^calculated, 185.2. ^1^H NMR (400 MHz, DMSO-*d_6_*) δ 6.29 (t, J = 2.0 Hz, 1H), 6.20 (dt, J = 10.8, 2.2 Hz, 1H), 6.14 (dt, J = 11.4, 2.2 Hz, 1H), 5.43 (bs, 2H), 3.09 (s, 3H), 1.39 (s, 9H).

**tert-butyl (3-chloro-5-nitrophenyl) carbamate (7b, Scheme S2 – equation b).** The title compound was prepared according to general procedure 5 using nitroaniline **6b** (345.0 mg, 2.0 mmol), DIPEA (0.53 mL, 3.00 mmol), DMAP (50.0 mg, 0.40 mmol), and Boc_2_O (524 mg, 2.40 mmol) in DCM (10 mL). Final normal phase purification afforded pure compound **7b** (435.3 mg, 80% yield). UPLC/MS: Rt = 1.60 min (method 2), MS (ESI) m/z: 271.0 [M − H]^−^, C_11_H_12_ClN_2_O_4_ [M − H]^−^ calculated, 271.7. ^1^H NMR (101 MHz, DMSO-*d_6_*): δ 10.09 (s, 1H), 8.36 (t, J = 2.0 Hz, 1H), 7.88 (t, J = 1.9 Hz, 1H), 7.85 (t, J = 2.0 Hz, 1H), 1.50 (s, 9H).

**tert-butyl (3-chloro-5-nitrophenyl) (methyl)carbamate (8b, Scheme S2 – equation b).** The title compound was prepared according to general procedure 4 using compound **7b** (441.0 mg, 1.62 mmol), K_2_CO_3_ (447.0 mg, 3.24 mmol), iodoethane (0.24 mL, 3.24 mmol), in DMF dry (3.2 mL). Final normal phase purification (elution by gradient from 100% to 90/10 cyclohexane/EtOAc) afforded pure title compound **8b** (412.8 mg, 90% yield). UPLC/MS: Rt = 1.66 min (method 2), MS (ESI) m/z: 230.8 [M – ^t^Bu + H]^+^, C_8_H_8_ClN_2_O_4_ [M + H]^+^ calculated, 231.6. ^1^H NMR (400 MHz, DMSO-*d_6_*) δ 8.17 (t, J = 2.0 Hz, 1H), 8.05 (t, J = 2.0 Hz, 1H), 7.91 (t, J = 2.0 Hz, 1H), 3.27 (s, 3H), 1.44 (s, 9H).

**tert-butyl (3-amino-5-chlorophenyl) (methyl)carbamate (2e**). The title compound was prepared according to general procedure 3 using intermediate **8b** (210 mg, 073 mmol), Pd/C (42 mg), Et_3_SiH (1.74 mL, 10.95 mmol), in EtOH (3.3 mL), and reaction mixture stirred at 50°C for 5 hours. The reduction of substrate **8b** required modified conditions: increased loading of Pd/C and 15 equiv. of reducing agent. Final normal phase purification (elution by gradient from 100% to 60/40 cyclohexane/EtOAc) afforded pure title compound **2e** (116.2 mg, 62% yield). UPLC/MS: Rt = 1.00 min (method 2). MS (ESI) m/z: 257.0 [M + H]^+^, C_12_H_18_ClN_2_O_2_ [M + H]^+^calculated, 257.7. ^1^H NMR (400 MHz, DMSO-*d_6_*) δ 6.43 – 6.38 (m, 3H), 5.44 (s, 2H), 3.09 (s, 3H), 1.39 (s, 9H).

**NMR of final Topo-II inhibitors**

^1^H (400 MHz, DMSO-*d_6_*) of ARN-25656

^13^C (101 MHz, DMSO-*d_6_*) of ARN-25656

^1^H (400 MHz, DMSO-*d_6_*) of ARN-26009

^13^C (101 MHz, DMSO-*d_6_*) of ARN-26009

^1^H (400 MHz, DMSO-*d_6_*) of ARN-26010

^13^C (101 MHz, DMSO-*d_6_*) of ARN-26010

^1^H (400 MHz, DMSO-*d_6_*) of ARN-26672

^13^C (101 MHz, DMSO-*d_6_*) of ARN-26672

^1^H (400 MHz, DMSO-*d_6_*) of ARN-26673

^13^C (101 MHz, DMSO-*d_6_*) of ARN-26673
